# Contribution of V_H_ Replacement Products in Mouse Antibody Repertoire

**DOI:** 10.1371/journal.pone.0057877

**Published:** 2013-02-28

**Authors:** Lin Huang, Miles D. Lange, Yangsheng Yu, Song Li, Kaihong Su, Zhixin Zhang

**Affiliations:** 1 Department of Pathology and Microbiology, University of Nebraska Medical Center, Omaha, Nebraska, United States of America; 2 The Eppley Cancer Institute, University of Nebraska Medical Center, Omaha, Nebraska, United States of America; 3 Department of Internal Medicine, University of Nebraska Medical Center, Omaha, Nebraska, United States of America; Chang Gung University, Taiwan

## Abstract

V_H_ replacement occurs through RAG-mediated recombination between the cryptic recombination signal sequence (cRSS) near the 3′ end of a rearranged V_H_ gene and the 23-bp RSS from an upstream unrearranged V_H_ gene. Due to the location of the cRSS, V_H_ replacement leaves a short stretch of nucleotides from the previously rearranged V_H_ gene at the newly formed V-D junction, which can be used as a marker to identify V_H_ replacement products. To determine the contribution of V_H_ replacement products to mouse antibody repertoire, we developed a Java-based V_H_ Replacement Footprint Analyzer (V_H_RFA) program and analyzed 17,179 mouse IgH gene sequences from the NCBI database to identify V_H_ replacement products. The overall frequency of V_H_ replacement products in these IgH genes is 5.29% based on the identification of pentameric V_H_ replacement footprints at their V-D junctions. The identified V_H_ replacement products are distributed similarly in IgH genes using most families of V_H_ genes, although different families of V_H_ genes are used differentially. The frequencies of V_H_ replacement products are significantly elevated in IgH genes derived from several strains of autoimmune prone mice and in IgH genes encoding autoantibodies. Moreover, the identified V_H_ replacement footprints in IgH genes from autoimmune prone mice or IgH genes encoding autoantibodies preferentially encode positively charged amino acids. These results revealed a significant contribution of V_H_ replacement products to the diversification of antibody repertoire and potentially, to the generation of autoantibodies in mice.

## Introduction

The variable region exons of the immunoglobulin (Ig) genes are generated through sequential rearrangement of previously separated V_H_, D_H_ (for heavy chain only), and J_H_ gene segments catalyzed by the recombination activating gene products (RAG1 and RAG2) [1]–[5]. The specific joining of V_H_, D_H_, and J_H_ gene segments is directed by the recombination signal sequences (RSSs) [6], [7]. The RSS consists of a highly conserved heptamer and a nonamer, separated by a non-conserved spacer region with either 12-bp or 23-bp nucleotides [6]–[9]. Efficient recombination occurs between a 12 bp RSS- and a 23 bp RSS-flanked gene segments [6], [7]. After RAG-mediated cleavage, the resulting double strand DNA breaks are repaired by the Non-Homologous End Joining (NHEJ) pathway [4], [5]. The coding end hairpins are opened and re-joined to form the coding exon of Ig gene, whereas the signal ends are ligated to form an excision circle and released from the chromosomal DNA [6], [7].

Rearrangement of Ig heavy (IgH) chain genes starts with a D_H_ to J_H_ recombination on one allele of the IgH loci in early progenitor (pro) B cells followed by recombining a V_H_ gene segment to the DJ_H_ joint in late pro B cells [4], [5]. If the rearrangement is non functional, pro B cells will start to rearrange the second IgH allele [4], [5]. Functionally rearranged IgH genes will be expressed as the µ heavy chains to form pre-B cell receptors with the non-rearranged components, Vpre-B and lambda 5 [10]–[15]. Signaling from the pre-BCR will stimulate pre B cell proliferation and subsequent IgL gene rearrangement [14], [15]. The IgL gene variable region exon is generated by a one step rearrangement between a V_L_ segment and a J_L_ segment in the small precursor (pre-) B cells [4], [5], [16]. Due to the random recombination process, two thirds of the V(D)J rearrangement products might be out of reading frame and cannot express functional Ig peptides. Even if the IgH gene rearrangements are productive, they might fail to pair with the surrogate or conventional light chains. B cells lacking functional pre-B cell receptors (pre-BCRs) or B cell receptors (BCRs) cannot develop further along the B lineage pathway [14], [17]. Moreover, functionally expressed BCRs may be self-reactive. In all these cases, early B lineage cells retain the abilities to initiate secondary RAG-mediated recombination to alter the rearranged Ig genes, a process known as receptor editing [18]–[20].

Editing of rearranged IgL genes can occur through RAG-mediated secondary recombination between any upstream V_L_ gene to a downstream J_L_ gene [21]–[26]. The intervening DNA fragment containing the previously rearranged V_L_J_L_ joint is deleted during the editing process [24]–[26]. As a default mechanism, pre-B cells with non-functional rearrangements on both *Igκ* alleles can initiate *de novo* rearrangements at the *Igλ* locus [26]. Accumulating studies indicated that non-functional or autoreactive IgH gene rearrangements can be edited through a V_H_ replacement process [27]–[33]. V_H_ replacement occurs through RAG-mediated recombination between a cryptic RSS embedded at the 3′ end of the rearranged V_H_ gene with the 23 bp RSS from a upstream V_H_ gene [31]. V_H_ replacement was originally observed in murine pre-B cell leukemia cells, which generated functional IgH genes from non-functional IgH rearrangements [27], [28]. The potential biological function of V_H_ replacement in editing IgH genes encoding anti-DNA antibodies was demonstrated in a series of studies using engineered mouse models carrying knocked-in IgH V(D)J rearrangements encoding anti-DNA antibodies [29], [34], [35]; Later studies also provided evidence that V_H_ replacement was employed to diversity the antibody repertoire in mouse carrying knocked-in IgH genes encoding anti-NP antibodies [30], [36] and to rescue B cells with two alleles of non-functional IgH rearrangements [32], [33]. Despite of these findings in engineered mice, evidence for ongoing V_H_ replacement during B cell development in normal mouse and contribution of V_H_ replacement products to the mouse antibody repertoire were lacking for a long time [37], [38].

Due to the location of the cRSS at the 3′ end of V_H_ germline gene, V_H_ replacement renews almost the entire V_H_ coding region but leaves a short stretch of nucleotides from the previously rearranged V_H_ gene at the newly formed V-D junction [28], [31]. These remnants can be used as V_H_ replacement footprints to trace the occurrence of V_H_ replacement and to identify potential V_H_ replacement products through analyzing IgH gene sequences [31]. Our previous analysis of 412 human IgH gene sequences estimated that V_H_ replacement products contribute to about 5% of the primary B cell repertoire in human [31]. A recent analysis of IgH genes generated from knock-in mice expressing IgH genes encoding anti-DNA antibodies showed that 7.5% of the newly generated IgH genes contain pentameric V_H_ replacement footprints [39]. Similar frequency of V_H_ replacement products were also found in IgH genes obtained from the wild type B6 mice [39].

To explore the contribution of V_H_ replacement products to the diversification of mouse IgH repertoire, we developed a Java based V_H_ replacement footprint analyzer (V_H_RFA) program and analyzed 17,179 mouse IgH gene sequences from the National Center for Biotechnology Information (NCBI) database to identify V_H_ replacement products. These results revealed a significant contribution of V_H_ replacement products to the murine IgH repertoire and the enrichment of V_H_ replacement products in several strains of autoimmune prone mice.

## Results

### The Mouse IgH Sequence Repertoire

**Figure 1:**
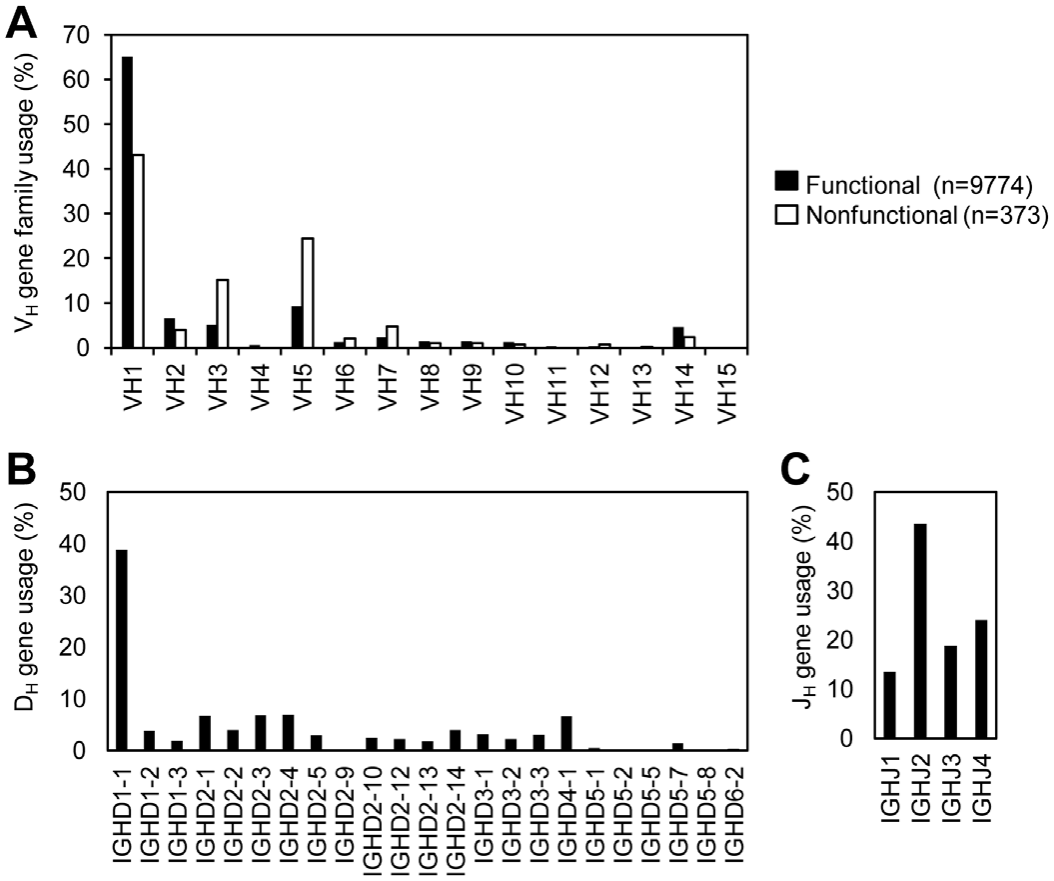
Immunoglobulin VH, DH, and JH gene usages in the mouse IgH sequence repertoire. The mouse IgH gene sequence data set containing 17,179 entries was downloaded from NCBI databases. The potential V_H_, D_H_, and J_H_ germline gene assignments were performed using the IMGT/V-QUEST program by sending batches of sequences by the V_H_RFA program. Clonally redundant IgH sequences were removed if they contain identical CDR3 regions. The usages of different families of V_H_ germline genes (A), D_H_ genes (B), and J_H_ (C) genes in the functional or non-functional unique IgH genes were analyzed.

To analyze a large number of IgH gene sequences and to identify potential V_H_ replacement products, we developed a Java based V_H_ Replacement Footprint Analyzer (V_H_RFA) program. Using the V_H_RFA program, we analyzed 17,179 mouse IgH gene sequences from the NCBI databases to identify V_H_ replacement products. First, the potential V_H_, D_H_, and J_H_ germline gene usage were assigned using the IMGT/V-QUEST program by sending batches of sequences using the V_H_RFA program (shown in Table S1). Based on the IgH CDR3 region sequences, clonally identical sequences were stripped out. There are 11309 unique IgH gene sequences; 10159 of them have clearly identifiable V_H_, D_H_, and J_H_ genes; 9774 of them are productive and 373 of them are non-productive IgH rearrangements. In these IgH genes, different families of V_H_ genes are used differentially (Fig. 1). There are 63683 (65%) functional IgH genes using the IGHV1/V_H_J558 family of V_H_ genes; 911 (or 9.3%) functional IgH genes using the IGHV5/V_H_7183 family of V_H_ genes. The other families of V_H_ genes, including IGHV4/X-24, IGHV11/CP3, IGHV12/CH27, IGHV13/3609N, and IGHV15/VH15A, are used at much lower frequencies (Fig. 1A). Among the non-functional IgH rearrangements, the usages of most V_H_ gene families are similar to those in functional IgH genes, but the usages of the IGHV5/V_H_7183 and IGHV3/36–60 gene families are increased (Fig. 1A). Among different D_H_ genes, the IGHD1-1 gene is used the most frequent in almost 39% of the IgH sequences (Fig. 1B). For the J_H_ genes, the IGHJ2 gene is used the most frequent in 43% of IgH genes (Fig. 1C). It should be noted that these 17179 mouse IgH sequences were derived from about 861 published reports (Table S2), presumably from more than 861 experiments with different mice. This analysis represents a comprehensive view of the IgH repertoire of the current available mouse IgH gene sequences in the NCBI database.

### Identification of V_H_ Replacement Products

In the initial test, we use the V_H_RFA program to identify potential V_H_ replacement products in 271 mouse IgH gene sequences described previously [40]. Among them, 252 unique IgH genes have clearly identifiable V_H_, D_H_, and J_H_ germline genes. Then, we searched for V_H_ replacement footprint motifs with 3, 4, 5, 6, or 7 nucleotides within the V_H_-D_H_ junction (N1) regions of these IgH genes. V_H_ replacement can only introduce V_H_ replacement footprint in the N1 region. As an internal control, we searched for similar V_H_ replacement footprint motifs in the D_H_-J_H_ junction (N2) regions of these IgH genes, which are likely generated by random nucleotide addition. The frequencies of 3, 4, and 5-mer V_H_ replacement footprint motifs in the N1 regions are significantly higher than those in the N2 regions (Table 1, top), suggesting that the distribution of such motifs in the N1 region is not due to random nucleotide addition. Based on the identification of the pentameric V_H_ replacement footprints within the N1 regions, we estimate that the frequency of V_H_ replacement products is 5.5% in these 252 mouse IgH gene sequences (Table 1, Top). If we consider the 4- or 3-mer of V_H_ replacement footprints in the N1 regions, the frequencies of V_H_ replacement products in these 252 IgH genes will be 21.2% or 38%, respectively (Table 1, top and the identified V_H_ replacement products with 4-mer V_H_ replacement footprints are shown in Table S5).

**Table 1:** Frequencies of V_H_ replacement footprint motifs with different length in the N1 and N2 regions of the mouse IgH genes.

	Number of unique Sequences^a^	Number of Sequences with V, D, J genes^b^	Minimal Length of V_H_ replacement footprint	V_H_ replacement footprint motifs in the N1 region^c^	V_H_ replacement footprint motifs in the N2 region^d^	*p*-value^e^	Frequency of V_H_ replacement products (%)^f^
Test IgH genes^g^	271	252	3	101	65	0.0001	40.1
			4	55	23	0.0001	21.8
			5	14	4	0.0308	5.5
			6	2	0	0.4786	0.79
			7	1	0	0.3168	0.39
NCBI IgH genes^h^	11309	10159	3	3384	2622	0.0001	33.55
			4	1609	979	0.0001	15.95
			5	534	256	0.0001	5.29
			6	179	50	0.0001	1.77
			7	45	8	0.0001	0.45

a Unique sequences were identified after removal of IgH sequences with identical CDR3 regions.

b Total number of IgH gene sequences with clearly identifiable V_H_, D_H_, and J_H_ genes.

c N1 region refers to the V-D junction.

d N2 region refers to the D-J junction.

e The frequencies of potential V_H_ replacement footprint motifs in the N1 and N2 regions were compared by two-tailed Chi-square with Yate’s correction. *p*<0.05 was considered significant and *p*<0.0001is considered extremely significant.

f Numbers of IgH gene sequences with V_H_ replacement “footprint” motifs in the N1 regions were divided by the total number of IgH gene sequences with V, D, J gene assignment.

g Mouse IgH gene sequences were previously described.

h The mouse IgH gene sequences were downloaded from the NCBI database on May 7, 2011. The GI numbers of these sequences were included in Table S1.

Further analysis of the 14 identified V_H_ replacement products validated the assignment of V_H_ replacement footprints by the V_H_RFA program (Table 2). Theoretically, V_H_ replacement occurs through an upstream V_H_ gene replacing a downstream rearranged V_H_ gene. Among these 14 identified potential V_H_ replacement products, 11 of them were likely generated through upstream V_H_ genes replacing downstream V_H_ genes; 3 of them did not follow such order (Table 2).

**Table 2:** List of potential V_H_ replacement products in the test IgH sequences.

Sequence ID	V_H_ gene	3′ V_H_	P	N1	D_H_	Potential footprint donor	Position^a^
FJ816520	IGHV1S132	tgtgcaaga		gggaggacct	IGHD2-14	IHGV8-10, IGHV8-14, IGHV8S2	Y
FJ150867	IGHV14-3	tgtgcaaga		gggagaggggggcgtgatc	IGHD1-1	IGHV3-3, IGHV10-3, IGHV13-1	Y
FJ150854	IGHV1S132	tgtgcaaga		gcgaacg	IGHD2-12	IGHV7-1	Y
GU907018	IGHV1-9	tgtgccaga		ggagga	IGHD1-1	IGHV8-10, IGHV8-14, IGHV8S2	Y
FJ816537	IGHV1-74	tgtgcaa		gagagg	IGHD2-12	IGHV3-3, IGHV10-3, IGHV13-1	Y
FJ816495	IGHV1-47	tgtgcaagg		gagag	IGHD1-1	IGHV3-3, IGHV10-3, IGHV13-1	Y
GU907010	IGHV1-5	tgtacaaga		gagac	IGHD2-1	IGHV10-1, IGHV12-3	Y
GU907038	IGHV1-4	tgtgcaaga	tc	gaagg	IGHD2-3	IGHV3-1	Y
FJ816546	IGHV1-4	tgtgcaag		gaagagg	IGHD1-1	IGHV8-12, IGHV1-11, IGHV12-3	Y
FJ816592	IGHV14-1	tgtgc		cagag	IGHD2-14	IGHV2-6-7	Y
FJ816442	IGHV14-1	tgtgcta		aaacctc	IGHD1-1	IGHV2-3, IGHV2-6-6	Y
FJ816522	IGHV2-9-1	tgtgccagaga	tc	ggggatatcg	IGHD2-14	IGHV7-3	N
GU906999	IGHV14-3	tgtgctaga		ggagga	IGHD1-1	IGHV8-10, IGHV8-14, IGHV8S2	N
GU906995	IGHV14-3	tgtgctgga		ggagga	IGHD1-1	IGHV8-10, IGHV8-14, IGHV8S2	N

The identified V_H_ replacement footprints in the N1 regions are *underlined*.

a The relative positions of the potential donors and recipient V_H_ genes in the identified V_H_ replacement product were analyzed to determine if the V_H_ replacement occurred through an upstream V_H_ gene replacing a downstream V_H_ gene (Y) or a downstream V_H_ gene replacing an upstream gene (N). Only functional V_H_ germline genes were used in this analysis.

### Contribution of V_H_ Replacement Products to the Mouse IgH Repertoire

Next, we analyzed the 11,309 unique mouse IgH gene sequences from the NCBI database using the V_H_RFA program to search for V_H_ replacement products. We performed separated analyses to identify V_H_ replacement footprints with 3, 4, 5, 6, and 7 nucleotides in the V_H_-D_H_ junction (N1) regions. As internal controls, we also searched for the similar motifs in the D_H_-J_H_ junction (N2) regions. The frequencies of identified V_H_ replacement footprints with 3, 4, 5, 6, or 7 nucleotides in the N1 regions are significantly higher than those in the N2 regions (Table 1, bottom). These results indicate that the presence of these motifs at the N1 region is not due to random nucleotide addition. With a stringent setting to search for the pentameric V_H_ replacement footprints at the N1 regions, 5.29% of the IgH genes contain such motifs and can be assigned as potential V_H_ replacement products. If we consider V_H_ replacement footprints with 4 or 3 nucleotides, 15.95% or 33.55% of the IgH genes, respectively, contain such motifs and can be assigned as potential V_H_ replacement products (Table 1, bottom). These results revealed a significant contribution of V_H_ replacement products to the diversification of the murine IgH repertoire.

### Distribution of V_H_ Replacement Products in IgH Genes Using Different Families of V_H_ Genes

As we showed earlier, different V_H_ gene families are used at different frequencies in the 10159 mouse IgH gene sequences. Next, we analyzed the distribution of the identified V_H_ replacement products with 5-mer footprint motifs in IgH genes using different V_H_ gene families. Among all the IgH genes using different families of V_H_ genes, the frequency of V_H_ replacement products in IgH genes using the VH2/Q52 genes is significantly higher than that in the overall mouse IgH sequences (Table 3). The frequencies of V_H_ replacement products in IgH genes using the other V_H_ gene families are quite similar. For example, although the IGHV1/V_H_J558 and IGHV5/V_H_7183 families are used most frequently and the IGHV4/X-24, IGHV12/CH27, and IGHV14/SM7 families are used at very low frequencies, the frequencies of V_H_ replacement products in IgH genes using the IGHV1/V_H_J558, IGHV5/V_H_7183, IGHV4/X-24, IGHV12/CH27, and IGHV14/SM7 families are similar (Table 3). These results indicate that although different families of V_H_ genes are used differentially during the primary V(D)J recombination, they are similarly targeted for secondary recombination during V_H_ replacement. As an internal negative control, we analyzed the N1 regions of IgH genes using the D_H_ proximal V_H_5-2/7183.2 gene. Among the 56 functional IgH genes using the V_H_5-2/7183.2 gene, there is no pentameric V_H_ replacement footprints in the N1 regions. Such result provides supporting evidence that the presence of pentameric footprints in the N1 regions of mouse IgH genes is contributed by V_H_ replacement.

**Table 3:** Frequencies of V_H_ replacement products in IgH genes using different families of mouse V_H_ genes.

V_H_ family	Number of IgH gene sequences	Motifs in the N1 region	Frequency of V_H_ replacement products (%)^a^
VH1/J558	6530	314	4.81
VH2/Q52	665	55	8.27^c^
VH3/36-60	565	30	5.31
VH4/X-24	57	3	5.26
VH5/7183	998	68	6.81
VH6/J606	131	6	4.58
VH7S107	253	8	3.16
VH8/3609	139	9	6.47
VH9/VGAM3-8	144	11	7.64
VH10/VH10	127	4	3.15
VH11/CP3	37	0	0
VH12/CH27	43	3	6.98
VH13/3609N	7	1	14.29
VH14/SM7	459	26	5.66
VH15/VH15A	4	0	0
VH5-2/7183.2^b^	56	0	0

a Number of IgH gene sequences with V_H_ replacement “footprint” motifs in the N1 regions divided by the total number of IgH gene sequences assigned to a V_H_ gene family.

b Functional IgH genes using the VH5-2/7183.2 gene were analyzed for potential V_H_ replacement footprints in the N1 regions.

C The frequency of V_H_ replacement products using VH2/Q52 family of V_H_ genes is significantly higher than the overall frequency of V_H_ replacement products in mouse IgH genes.

### Enrichment of V_H_ Replacement Products in IgH Genes Derived from Different Strains of Autoimmune Prone Mice and IgH Genes Encoding Autoantibodies

**Figure 2:**
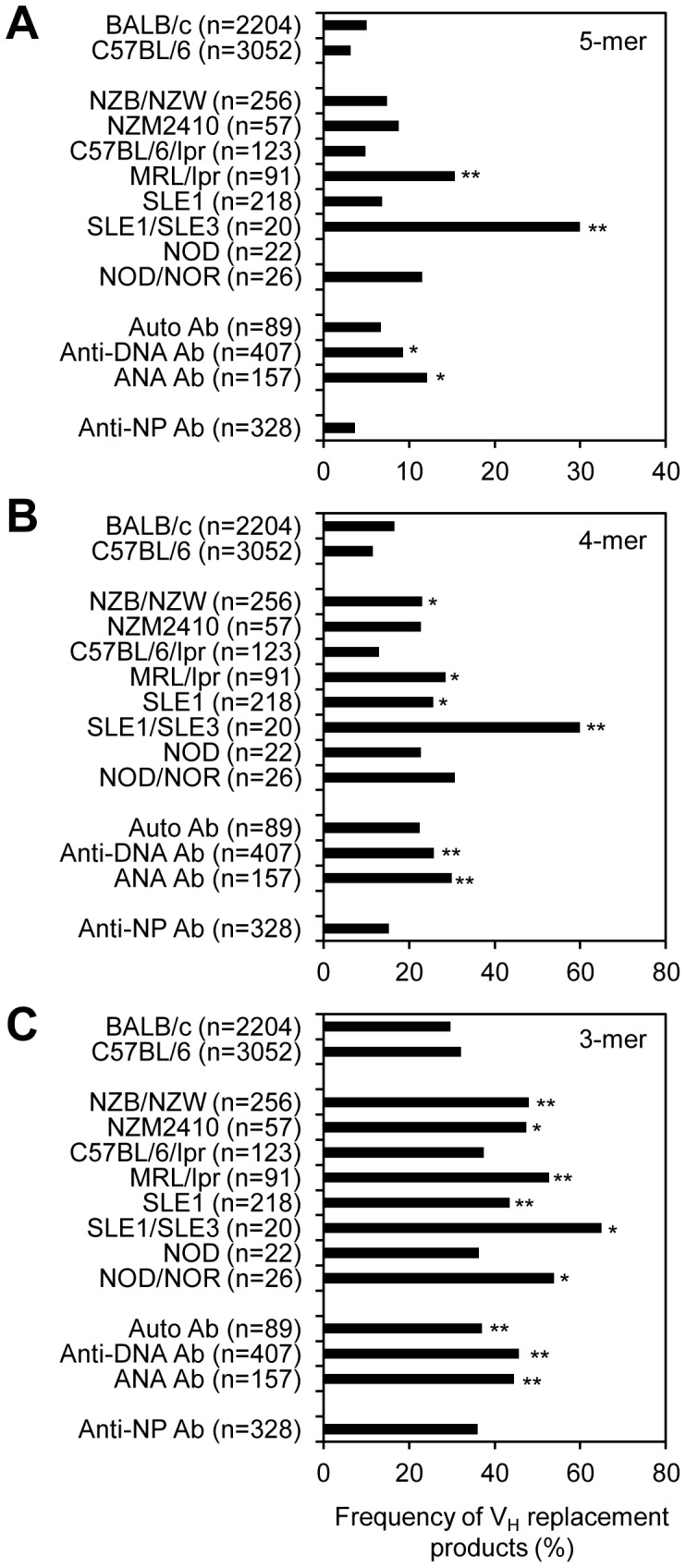
Enrichment of V_H_ replacement products in IgH genes derived from different strains of autoimmune prone mice and IgH genes encoding autoantibodies. The frequencies of V_H_ replacement products in IgH genes derived from different strains of mice were analyzed using the V_H_RFA program based on the keyword linked to each IgH gene in the NCBI database. V_H_ replacement products were assigned based on the identification of (A) 5-mer V_H_ replacement footprints, (B) 4-mer V_H_ replacement footprints, or (C) 3-mer V_H_ replacement footprints within the V_H_-D_H_ junctions (N1 regions). The frequencies of V_H_ replacement products in different subcategories were compared with that in the *BALB/c* mice. *n*, number of IgH sequences in each subcategory. Statistical significance was determined using a two-tailed Chi square test with Yate’s correction. *p*<0.05 (*) is considered significant and *p*<0.0001 (**) is considered extremely significant. The detailed sequence analysis and the identified V_H_ replacement products with 5-mer V_H_ replacement footprints correlating with keywords are included in Table S6.

To explore the biological significance of V_H_ replacement in mouse, we analyzed the distribution of V_H_ replacement products in IgH genes correlating with different keywords in the NCBI database. Based on the identification of 5-mer V_H_ replacement footprints within the N1 regions, the frequencies of V_H_ replacement products in IgH genes derived from *C57BL/6* and *BALB/c* strains of mice are 3.17% and 5%, respectively (Fig. 2A and Table S6). Such numbers may serve as the basal levels of V_H_ replacement products in these mice. Comparing IgH genes derived from several strains of mice, the frequencies of V_H_ replacement products are highly elevated in IgH genes derived from different strains of autoimmune prone mice (Fig. 2A). In particular, the frequencies of V_H_ replacement product are elevated in IgH genes derived from lupus prone *NZB/NZW* F1, *NZM2410*, *MRL/lpr*, and *SLE1/SLE3* mice. In IgH genes derived from mice carrying the spontaneous Fas^lpr^ mutation (*MRL/MpJ-Lpr/Lpr*), the frequency of V_H_ replacement products is 15.38%. In IgH genes from the *Sle1/Sle3* mice, the frequency of V_H_ replacement products is 30%. These frequencies are significantly higher than that in the *BALB/c* or *C57BL/6* mice (*p*<0.05, two tailed *Chi*-square test) (Fig. 2A). The elevated levels of V_H_ replacement products in autoimmune prone mice suggest that V_H_ replacement products contribute to the generation of autoantibodies. Indeed, further analyses of the IgH genes encoding different antibodies showed that the frequencies of V_H_ replacement products are 12.1% in IgH genes encoding ANA antibody and 9.34% in IgH genes encoding anti-DNA antibodies. These levels are significantly higher than those in the *BALB/c* or *C57BL/6* mice. As a negative control, the frequency of V_H_ replacement products in IgH genes obtained from mice immunized with NP is 3.66%, which is similar to that in the *C57BL/6* mice. Taken together, these results provide the first information that V_H_ replacement products are highly enriched in IgH genes derived from different strains of autoimmune prone mice and in IgH genes encoding anti-DNA and ANA autoantibodies.

Using the V_H_RFA program, we also analyzed the frequencies of V_H_ replacement products based on the 4- or 3-mer of V_H_ replacement footprints in IgH genes derived these diseased subcategories. Extending the assignment of V_H_ replacement products with considering the 4- and 3-mer V_H_ replacement footprints clearly increases the frequencies of V_H_ replacement products in IgH genes from all subcategories. With considering the 4-mer V_H_ replacement footprints, the frequencies of V_H_ replacement products in IgH genes derived from *NZB/NZW*, *MRL/lpr*, *SLE1*, *SLE1/SLE3* and IgH genes encoding anti-DNA and ANA antibodies are significantly higher than that in the *BALB/c* mice (*p*<0.05, two tailed *Chi*-square test) (Fig. 2B); with considering the 3-mer V_H_ replacement footprints, the frequencies of V_H_ replacement products in IgH genes derived from *NZB/NZW*, *NZM2410*, *MRL/lpr, SLE1*, *SLE1/SLE3*, *NOD/NOR* and IgH genes encoding auto antibodies, anti-DNA antibodies, and ANA antibodies are significantly higher than that in the *BALB/c* mice (*p*<0.05, two tailed *Chi*-square test) (Fig. 2C). Taken together, these results showed that V_H_ replacement products are enriched in IgH genes derived from different strains of autoimmune prone mice and in IgH genes encoding autoantibodies.

### The Identified V_H_ Replacement Footprints Preferentially Encode Charged Amino Acids

**Figure 3:**
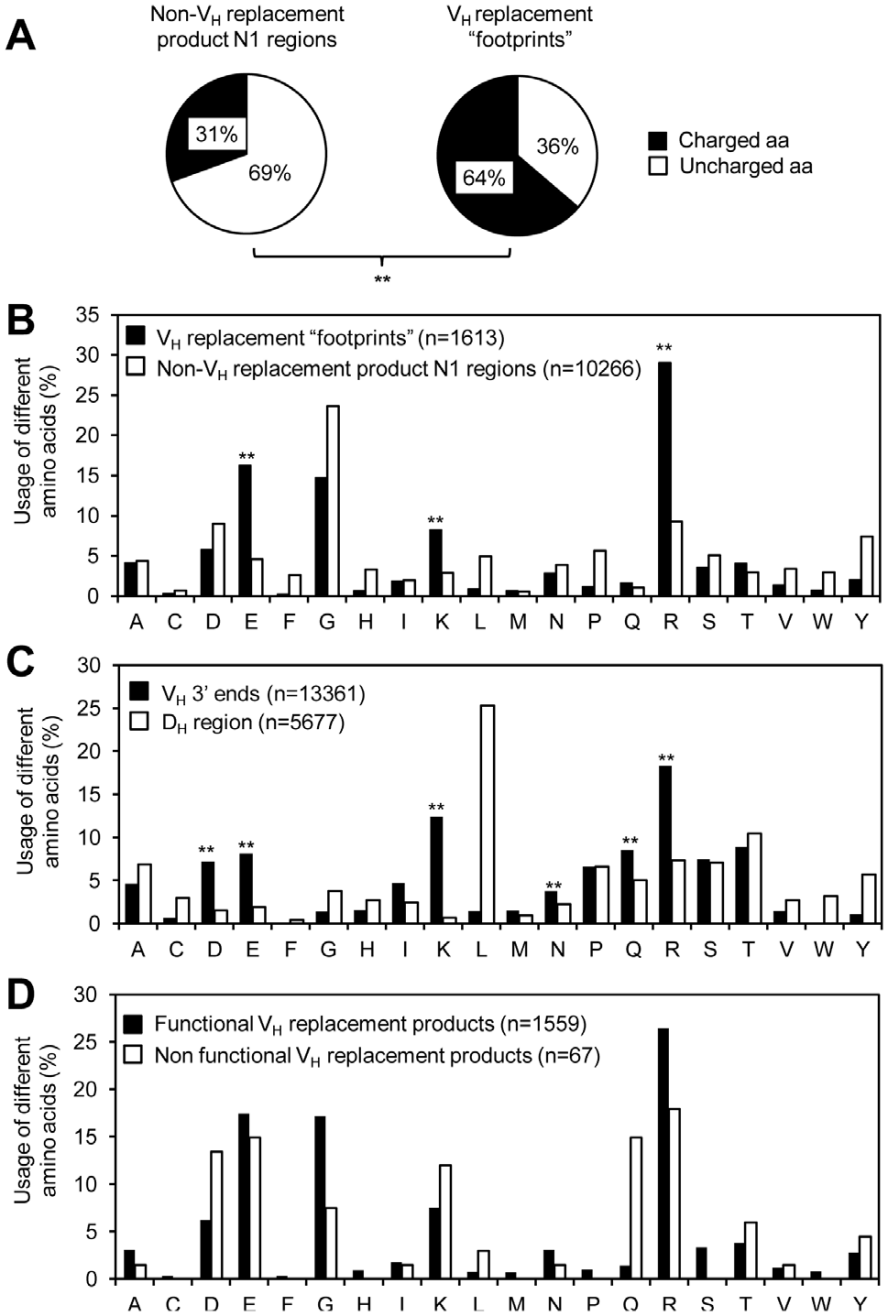
VH replacement footprints preferentially contribute charged amino acids to the CDR3 regions. (A) The frequencies of charged amino acids encoded by the identified pentameric V_H_ replacement footprints or the N1 regions of non-V_H_ replacement products were compared. Detailed amino acid sequences of the IgH CDR3 regions are listed in Table S6. (B) The frequencies of individual amino acid encoded by the identified V_H_ replacement footprints or the N1 regions of non-V_H_ replacement products were compared. *n*, amino acids encoded by the identified V_H_ replacement footprints or the N1 regions of non-V_H_ replacement products. (C) The frequencies of individual amino acid encoded by the 3′ end of V_H_ germline genes and D_H_ regions were compared. n, amino acids encoded by the V_H_ gene 3′ ends or D_H_ regions. (D) Usages of different amino acids encoded by the identified V_H_ replacement footprints in functional V_H_ replacement products and non-functional V_H_ replacement products. n, amino acids encoded by the identified V_H_ replacement footprints. Statistical significance was determined using a two-tailed Chi square test with Yate’s correction. *n*, number of amino acid residues encoded by indicated sequences. *p*<0.05 (*) is considered significant and *p*<0.0001 (**) is considered extremely significant.

Our previous analysis of the identified V_H_ replacement products in human IgH genes showed that the V_H_ replacement footprints preferentially encode charged amino acids into the IgH CDR3 regions [31]. Here, analysis of the identified V_H_ replacement products from mouse IgH genes showed that 64% of the amino acids encoded by the identified V_H_ replacement footprints contribute charged amino acids, including K, R, D, E, N, and Q. Such frequency is significantly higher than the overall frequency of charged amino acids in the N1 regions (*p*<0.0001) (Fig. 3A). Moreover, the frequencies of charged amino acids, including E, K, and R, encoded by the identified V_H_ replacement footprints are significantly higher than those encoded by the N1 regions of non-V_H_ replacement products (*p*<0.0001) (Fig. 3B). The preferential contribution of charged amino acids by the V_H_ replacement footprints seems to be predetermined by the sequences at the 3′ end of V_H_ germline genes following the cRSS sites. The frequencies of charged amino acids encoded by the 3′ ends of V_H_ germline gene, including K, R, D, E, N, and Q, are significantly higher than those encoded by the D_H_ germline genes (*p*<0.0001) (Fig. 3C). In non-functional IgH genes, the identified V_H_ replacement footprints also preferentially encode charged amino acids, although the usages of different charged residues are slightly different from those in the functional V_H_ replacement products (Fig. 3D). Such results are consistent with previous findings that the V_H_ replacement footprints identified in human or mouse V_H_ replacement products preferentially encoded charged residues [31], [39].

### The 3-mer V_H_ Replacement Footprints are Less Likely Contribute Charged Amino Acids to the CDR3 Regions

**Figure 4:**
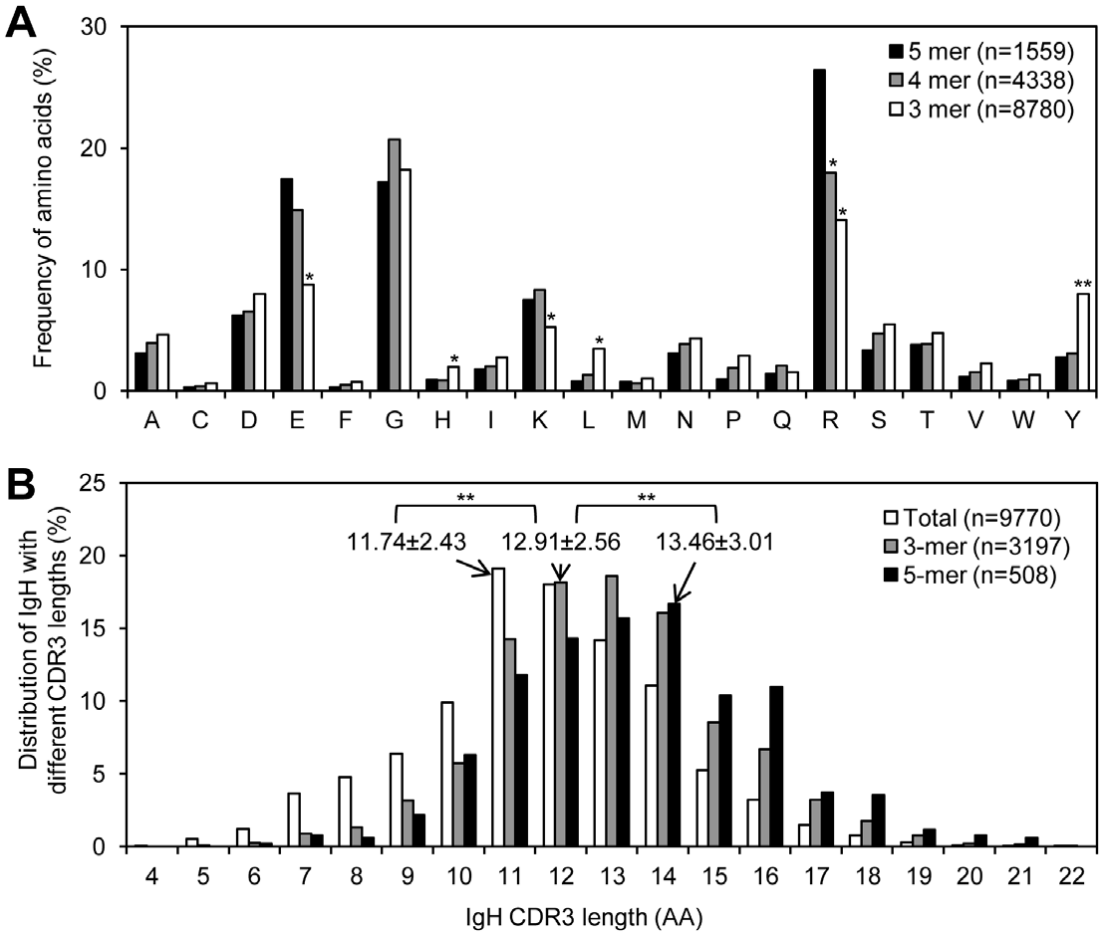
Comparison of the amino acids encoded by V_H_ replacement footprints and the IgH CDR3 lengths of V_H_ replacement products. (A) The usages of different amino acids encoded by V_H_ replacement footprints with 5, 4, or 3 nucleotides were compared. *n*, number of amino acid residues encoded by the identified V_H_ replacement footprints with different lengths. Statistical significance was determined using a two-tailed Chi square test with Yate’s correction. *p*<0.05 (*) is considered significant and *p*<0.0001 (**) is considered extremely significant. (B) Comparison of the IgH CDR3 lengths of V_H_ replacement products containing the 5-mer or the 3-mer V_H_ replacement products with the CDR3 length of the total functional IgH genes. *n*, number of IgH sequences or V_H_ replacement products with 3- or 5-mer V_H_ replacement footprints. Statistical significance was determined using unpaired *t* test. *p*<0.05 (*) is considered significant and *p*<0.0001 (**) is considered extremely significant.

V_H_ replacement was considered as a receptor editing process to change non-functional IgH rearrangements or IgH genes encoding autoantibodies [29], [41]. Finding that the 5-mer V_H_ replacement footprints preferentially encoded charged amino acids, especially R and K residues, is contrast to the original goal of V_H_ replacement to eliminate autoreactive IgH genes. Because charged residues within the IgH CDR3 might contribute to autoreactivity. Interestingly, when we analyzed the amino acids encoded by the identified 3-mer V_H_ replacement footprints, the usages of charged residues, including R, K, and E, are significantly reduced; meantime, the usages of several neutral residues, including H, L, and Y, are significantly increased (Fig. 4A). These results showed that shorter V_H_ replacement footprints are less likely to encode charged residues.

### V_H_ Replacement Products have Longer CDR3 Lengths

During V_H_ replacement products, a short stretch of nucleotides from previously rearranged V_H_ genes were left within the newly generated V_H_-DJ_H_ junctions [31]. Comparison of the IgH CDR3 lengths of the identified V_H_ replacement products showed that the average CDR3 length of V_H_ replacement products with 5-mer footprints is significantly longer than that of V_H_ replacement products with 3-mer footprints; the average CDR3 length of V_H_ replacement products with 3-mer footprints is significantly longer than that of the total functional IgH genes in the NCBI database (*p*<0.0001, unpaired *t* test) (Fig. 4B). These results indicate that elongation of IgH CDR3 region is one of the intrinsic features of V_H_ replacement.

### Selection of V_H_ Replacement Footprints Encoding Positively Charged Residues in Autoantibodies

**Figure 5:**
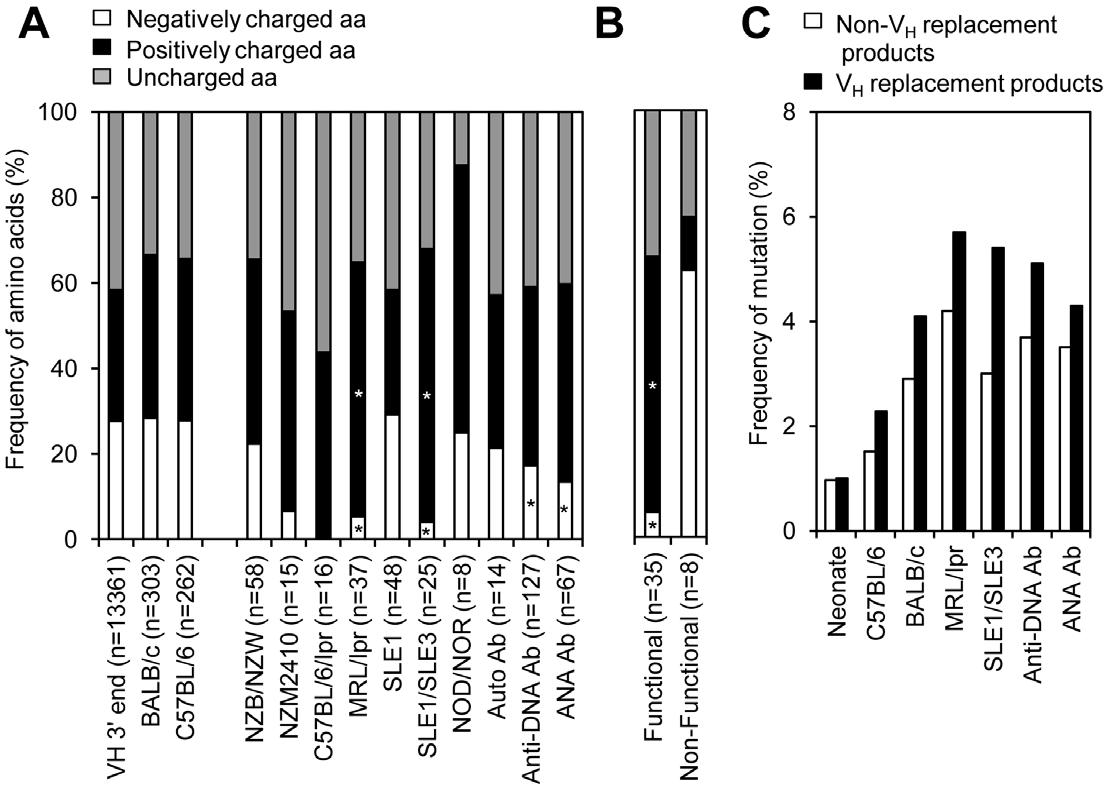
The enriched V_H_ replacement products identified in different strains of autoim*m*une prone mice or IgH genes encoding aut*o*antibodies have been positively selected during autoimmune responses. (A) Analysis of the frequencies of positively charged versus negatively charged amino acids encoded by the 3′ end V_H_ genes and the identified V_H_ replacement footprints from different strains of mice or IgH genes encoding autoantibodies. Statistical significance was determined using a two-tailed Chi square test with Yate’s correction. *p*<0.05 (*) is considered significant. (B) Comparison of the amino acids encoded by the identified V_H_ replacement footprints in *MRL/lpr* mice. *n*, numbers of amino acids encoded by the identified V_H_ replacement footprints. (C) Mutation status analysis of identified V_H_ replacement products and non-V_H_ replacement products from different subgroups of IgH genes.

The preferential contribution of charged amino acids by V_H_ replacement footprints is likely predetermined by the 3′ end sequences of V_H_ germline genes. Based on the 3′ end sequences of V_H_ germline genes, V_H_ replacement footprints can contribute almost equal numbers of positively or negatively charged residues (Fig. 5A). Indeed, in the identified V_H_ replacement products from IgH genes derived from *BALB/c* or *C57BL/6* mice, the frequencies of positively and negatively charged amino acids encoded by the V_H_ replacement products are similar (Fig. 5A). However, in the identified V_H_ replacement products in IgH genes from autoimmune prone mice, including *MRL/lpr and Sle1/Sle3* mice, the frequencies of positively charged residues encoded by the V_H_ replacement footprints are significantly higher than that in the control mice. Meantime, the frequencies of negatively charged residues encoded by the V_H_ replacement footprints are significantly lower than that in the control mice (Fig. 5A). The frequencies of negatively charged residues encoded by the identified V_H_ replacement footprints are significantly lower in IgH genes derived from *C56BL/6/lpr* mice and in IgH genes encoding anti-DNA or ANA antibodies (Fig. 5A). Detailed analysis of the functional versus non-functional IgH genes derived from *MRL/lpr* mice showed that the frequencies of positively charged residues encoded by the identified V_H_ replacement footprints were elevated in functional but not in non-functional IgH genes (Fig. 5B). These results indicate that the positively charged residues encoded by V_H_ replacement products were positively selected in these autoimmune prone mice.

### The Identified V_H_ Replacement Products are Mutated

The accumulation of V_H_ replacement products in IgH genes derived from different strains of autoimmune prone mice and IgH genes encoding different autoantibodies suggested that V_H_ replacement products contribute to the generation of autoantibodies in mice. Analyses of the mutation status of these identified V_H_ replacement products showed that the enriched V_H_ replacement products in autoimmune prone mice or IgH genes encoding anti-DNA or ANA autoantibodies are mutated (Fig. 5C), indicating that these V_H_ replacement products are positively selected in these autoimmune prone mice.

## Discussion

In the current report, we analyzed 17,179 mouse IgH gene sequences available from the NCBI database and provided a comprehensive view of the V_H_, D_H_, and J_H_ gene usages of these mouse IgH genes. Based on the identification of the pentameric V_H_ replacement footprints in the N1 regions, we estimated that the frequency of V_H_ replacement products in the 11309 unique mouse IgH gene sequences with identifiable D_H_ genes is 5.29%. Such result indicates a significant contribution of V_H_ replacement products to the diversification of murine antibody repertoire. This result is consistent with the previously estimated frequencies of V_H_ replacement products in human and mouse IgH genes [31], [39]. It should be pointed out that such estimation is based on the identification of V_H_ replacement footprints with a minimal length of 5 nucleotides. In comparison to human V_H_ germline genes, many mouse V_H_ germline genes have fewer nucleotides following the cRSS sites. Out of the 150 functional mouse V_H_ germline genes with cRSS sites, 60 of them have only 5 nucleotides following the cRSS sites. If there is any exo-nuclease activity to remove one nucleotide at either the 3′ or the 5′ end of the V_H_ replacement footprint during primary V_H_ to DJ_H_ recombination or V_H_ replacement recombination, respectively, the remaining V_H_ replacement footprints will have less than 5 nucleotides and cannot be identified from this analysis. Based on this consideration, assigning V_H_ replacement footprints with 4 or 3 nucleotides might be a reasonable and accurate method to identify potential V_H_ replacement products in mouse IgH genes. If we consider the 4- or 3-mer V_H_ replacement footprints at the N1 regions to assign V_H_ replacement products, the frequencies of V_H_ replacement products in the mouse IgH gene sequences should be 16% or 32%, respectively.

It has been shown previously that in mice carrying two non-functional alleles of IgH genes, V_H_ replacement occurs efficiently to generate almost normal number of B cells with a diversified repertoire [32], [33]. All these functional IgH genes in this mouse are generated through V_H_ replacement. However, only about 20% of the IgH gene sequences contain potential V_H_ replacement footprints (>3 mer). The other 80% of IgH gene sequences have no identifiable V_H_ replacement footprints [32], [33]. This result indicates that most of the V_H_ replacement footprints are deleted during V_H_ replacement recombination. Thus, even if using the minimal length of V_H_ replacement footprints with 4 or 3 nucleotides, we may still under-estimate the actual frequency of V_H_ replacement products in the murine IgH repertoire. Theoretically, 66.7% of the IgH rearrangements generated during V(D)J recombination will be out of reading frame and cannot produce functional IgH proteins; about 44% of the pro B cells undergoing V(D)J recombination should carry non-functional rearrangements on both IgH alleles. If V_H_ replacement can efficiently rescue these pro B cells, at least 44% of the expressed IgH genes should be generated by V_H_ replacement.

We should also point out that this sequence analysis based approach in identification of V_H_ replacement footprints may have false positive calls. Theoretically, there are no V_H_ replacement footprints in the N2 regions. In some of the IgH sequences, we identified similar 3, 4, or 5 mer V_H_ replacement footprint motifs in the N2 regions, although the frequencies of such motifs in the N2 regions are significantly lower than those in the N1 regions. The presence of such V_H_ replacement footprint motifs in the N2 regions could be due to random nucleotide addition during V(D)J recombination. In this regard, a low frequency of identified footprints might be false positive.

If we use the 5-mer V_H_ replacement footprints to assign V_H_ replacement products, the frequencies of V_H_ replacement products in IgH genes derived from *BALB/C* or *C57BL/6* mice are about 5% or 3.2%, respectively, which may represent the basal level of V_H_ replacement product in these two strains of mice. Interestingly, the frequencies of V_H_ replacement products are significantly elevated in IgH genes derived from different strains of autoimmune prone mice, including *MRL/Lpr* and *Sle1/Sle3* mice. It has been well demonstrated that these mice spontaneously produce anti-DNA or anti-ANA antibodies and develop lupus like symptom [42]–[49]. Indeed, V_H_ replacement products are significantly elevated in IgH genes encoding anti-DNA antibodies or ANA autoantibodies derived from mice with lupus glomerular nephritis. These results suggested a potential contribution of V_H_ replacement products to the generation of autoantibodies. When we consider the 4- or 3-mer V_H_ replacement footprints to assign V_H_ replacement products, the frequencies of V_H_ replacement products are elevated in all the sub-categories of IgH genes. Nevertheless, the frequencies of V_H_ replacement products in IgH genes derived from different strains of autoimmune prone mice and IgH genes encoding anti-DNA and ANA antibodies are significantly higher than that in the BALB/c mice.

Due to the location of the cRSS, V_H_ replacement will leave a short stretch of V_H_ replacement footprints to elongate the IgH CDR3 region [31], [41]. Strikingly, the identified pentameric V_H_ replacement footprints preferentially encode charged amino acids in the newly formed CDR3 regions. Such features are commonly found in V_H_ replacement products identified from human and mouse IgH genes [31], [39] and highly conserved in all the jawed vertebrates [50]. IgH genes with long CDR3 and charged residues are frequently encoding autoantibodies or anti-viral antibodies [51]. Here, our results showed that the frequencies of V_H_ replacement products are significantly elevated in IgH genes encoding anti-DNA and ANA autoantibodies in mouse. Theoretically, the V_H_ replacement footprints can encode either positively or negatively charged residues. Analysis of the amino acids encoded by the identified V_H_ replacement products from different strains of autoimmune prone mice and IgH genes encoding autoantibodies showed that the frequencies of positively charged residues encoded by V_H_ replacement footprints are significantly elevated; while the frequencies of negatively charged residues encoded by V_H_ replacement footprints are significantly reduced. Previous studies have shown that positively charged residue like Arg within the IgH CDR3 is critical for DNA binding [52]–[54]. These results suggested that the identified V_H_ replacement products from autoimmune prone mice have been positively selected. Such notion is also supported by the accumulated mutations in these identified V_H_ replacement products.

V_H_ replacement was originally recognized as a receptor editing process to change either non-functional IgH genes or IgH genes encoding autoreactive antibodies [20], [55]. The enrichment of V_H_ replacement products in IgH genes from different strains of autoimmune prone mice and in IgH genes encoding autoantibodies are surprising findings from this study. Currently, it is not clear why V_H_ replacement products are accumulated in autoimmune prone mice. Like any recombination process, V_H_ replacement is a random process that can generate non-functional IgH genes or IgH genes encoding autoreactive antibodies. Previous studies have shown that V_H_ replacement products generated through replacing the knocked-in anti-DNA IgH genes can produce high affinity anti-DNA antibodies during chronic graft-versus-host (cGVH) response [56]. Theoretically, after V_H_ replacement recombination, the newly generated IgH genes should be subjected to strict negative selection again to eliminate B cells expressing autoreactive BCRs. The observed accumulation of V_H_ replacement products in autoimmune prone mice could be due to the defective negative selection processes in these mice. In autoimmune prone mice, the newly generated V_H_ replacement products encoding autoreactive antibodies cannot be efficiently eliminated, but are rather positively selected and contribute to the generation of autoantibodies. To this extend, the different strains of autoimmune prone mice will be excellent experimental models to dissect how the V_H_ replacement products are selected and enriched during early B cell development.

Our analyses of the amino acid residues encoded by the identified V_H_ replacement footprints also uncovered an interesting finding that short V_H_ replacement footprints, especially the 3-mer footprints, encode less charged residues. These results suggested that if the V_H_ replacement footprints were trimmed down to 3-mer during either primary or secondary recombination, they will be less likely to contribute charged amino acids into the IgH CDR3 regions. Given the fact that 33.55% of IgH genes contain 3-mer V_H_ replacement footprints at their N1 regions, it is reasonable to conclude that the majority of these V_H_ replacement products successfully edited the IgH genes without introducing of extra charged residues into the newly formed CDR3 regions. The observed accumulation of V_H_ replacement products based on the identification of 5-mer footprints in the N1 regions in IgH genes derived from autoimmune prone mice may represent the failed V_H_ replacement attempts either due to defects in negative selection or defects in trimming down the V_H_ replacement footprints during primary or secondary recombination. Such findings raised several interesting questions that require further studies.

In conclusion, analysis of large number of mouse IgH gene sequences from the NCBI database provides a comprehensive view of the IgH repertoire of the available mouse IgH genes in the NCBI database and reveals a significant contribution of V_H_ replacement products to the diversification of mouse IgH repertoire. Identification of enriched V_H_ replacement products in IgH genes derived from different strains of autoimmune prone mice and IgH genes encoding autoantibodies indicated that abnormal regulation of V_H_ replacement may contribute to the generation of autoreactive antibodies.

## Materials and Methods

### Mouse IgH Sequences

Entrez IDs of mouse IgH sequences were provided by Igblast (http://www.ncbi.nlm.nih.gov/projects/igblast/) on May 07, 2011, which were used to download GenBank records of the sequences from NCBI. There were total 17,179 mouse IgH gene sequences retrieved at that time. The IDs of these IgH genes and their V_H_, D_H_, and J_H_ gene assignments are included in Table S1. After assignment of the potential germline V_H_, D_H_, J_H_ genes, clonally redundant sequences were stripped out based on their identical CDR3 regions. The resulting 11,308 unique sequences were further analyzed. Clonally related sequences with mutations within their CDR3 regions still remain. The 17179 mouse IgH sequences were derived from 861 published studies (Table S2). There were 1, 2, 4, 4, and 6 publications that contributed more than 500, 400–499, 300–399,200–299, and 100–199 sequences, respectively; 127 publications contributed 11–99 sequences; 717 publications contributed 10 or less than 10 sequences.

### The V_H_RFA Program

We developed a Java-based V_H_RFA program to incorporate assignments of the V_H_, D_H_, and J_H_ germline gene segments using the V-QUEST program (http://www.imgt.org/IMGT_vquest), identification of V_H_ replacement footprints with different lengths, analysis of amino acids encoded by the identified V_H_ replacement footprints, calculation of the amino acid usage encoded by the identified V_H_ replacement footprints, and correlation of the identified V_H_ replacement products with different keywords and publications associated with the sequences in the NCBI database.

### V_H_, D_H_, and J_H_ Germline Gene Assignment

Mouse IgH sequences in the GenBank format were converted to FASTA format and submitted to IMGT/V-QUEST (http://www.imgt.org/IMGT_vquest/share/textes/) for assign potential germline V_H_, D_H_, J_H_ genes, allowing 1 mutation at the 3′ end of V_H_ genes and at the 5′ end of J_H_ genes. All the IgH gene sequences were analyzed in batches containing 50 sequences each batch and the results were downloaded to a local computer as Excel files. These processes were conducted using the V_H_RFA program.

### Identification of V_H_ Replacement Footprint

All the rest steps were conducted on a local computer by the V_H_RFA program. First, a library file was generated, which contains all the potential V_H_ replacement footprints derived from functional V_H_ germline reference genes from the IMGT database (Table S3). Basically, the 3′ end segments following the cRSS sites from functional mouse V_H_ genes were sliced into different groups with 3, 4, 5, 6, 7, 8, 9, 10, and 11 nucleotides in length (Table S4). The V_H_RFA program will use this library to search the N1 (V_H_-D_H_ junction (N1) or D_H_-J_H_ junction (N2, as negative control) regions of the IgH genes to identify matched footprint motifs. For each IgH gene, the V_H_RFA program started by searching the longest footprint motifs (11 mer) from the 5′ to 3′ of the DNA sequences and then goes to search footprints with one nucleotide shorter. The identified footprints were listed if it does not overlap with any previously identified footprint within this region. For examples, the end results of footprint analyses of with specified 5 mer included all the footprints with 5, 6, 7, 8, 9, 10, and 11 mer from the V_H_ replacement footprint library. The end result was exported as a CVS file that contains the gene ID, functionality, V_H_, D_H_, J_H_ gene assignment, V_H_ replacement footprint in N1 (N1 signatures) or N2 (N2 signatures), together with other information from the original Excel file provided by the IMGT V-QUEST program. The identified footprints were shown in parenthesis within the N1 or N2 region sequences.

### Analysis of the Amino Acid Encoded by V_H_ Replacement Footprints, Keyword and Publication Linked to Each Gene, and Mutation

After identification of the V_H_ replacement footprints within the N1 regions, the V_H_RFA program further analyzed the amino acids encoded by the V_H_ replacement footprints and the usages of different amino acid. Each result was exported as an individual Excel file.

The V_H_RFA program can also analyze the original GenBank file to correlate the keywords and publication information with each IgH gene sequence. Basically, the V_H_RFA program parses the source GenBank file for keywords in the KEYWORDS and FEATURES sections of each entry sequence and output the keyword list in correlation with the sequence IDs, VDJ assignments, N1 footprints, and N2 footprints. Through this analysis, we can determine the distribution of V_H_ replacement products in different diseases.

For mutation analysis, the V_H_RFA program only calculated the mutation rate of IgH V_H_ genes with >80% similarities to the assigned germline V_H_ genes.

### Statistical Analysis

Statistical significance was determined by using either the two tailed *Chi*-square test with Yates’ correction or non paired student *t* test. Significant difference was determined if the *p* value <0.05.

## Supporting Information

Table S1 Analyses of mouse IgH genes and identification of VH replacement products.
(XLSX)

Table S2 Number of sequences from each publication.
(XLSX)

Table S3 Mouse VH genes containing the TACTGTG cRSS.
(DOCX)

Table S4 Potential mouse V_H_ replacement footprint motifs with different length.
(DOCX)

Table S5 Identification of 4-mer V_H_ replacement footprint motifs in mouse IgH sequences.
(DOCX)

Table S6 Identification of V_H_ replacement products in IgH genes correlating with different keywords.
(DOCX)
