# Supplementary material for: Contribution of VH Replacement Products in Mouse Antibody Repertoire
Source: PLoS One. 2013 Feb 28;8(2):e57877. doi: 10.1371/journal.pone.0057877 (PMC3585286; doi:10.1371/journal.pone.0057877)
Supplement: Table S3 — Mouse VH genes containing the TACTGTG cRSS. (DOCX) [file pone.0057877.s003.docx]

| **Table S3. Mouse V_H_ genes containing the TACTGTG cRSS.** | | | |
| --- | --- | --- | --- |
| **V_H_ Gene Name** | **3' sequence with cRSS** | **Sequences after cRSS** | **Functionality** |
| IGHV1-4*01 | TACTGTGCAAGA | CAAGA | F |
| IGHV1-4*02 | TACTGTGCAAGA | CAAGA | F |
| IGHV1-5*01 | TACTGTACAAGA |  | F |
| IGHV1-7*01 | TACTGTGCAAGA | CAAGA | F |
| IGHV1-8*01 | TACTGTACAAG |  | P |
| IGHV1-9*01 | TACTGTGCAAGA | CAAGA | F |
| IGHV1-11*01 | TACTGTGGAAGAGG | GAAGAGG | F |
| IGHV1-12*01 | TTCTGTGCAAGA |  | F |
| IGHV1-13*01 | TATTGTGAGAGA |  | P |
| IGHV1-14*01 | TACTGTGCAAGA | CAAGA | F |
| IGHV1-15*01 | TACTGTACAAGA |  | F |
| IGHV1-16*01 | TACTGTGCAAGA | CAAGA | ORF |
| IGHV1-17-1*01 | TACTGTGCAAGA | CAAGA | F |
| IGHV1-18*01 | TACTGTGCAAGA | CAAGA | F |
| IGHV1-18*02 |  |  | [F] |
| IGHV1-18*03 |  |  | [F] |
| IGHV1-19*01 | TACTGTGCAAGA | CAAGA | F |
| IGHV1-19-1*01 | TACCGTGCAAGA |  | P |
| IGHV1-20*01 | TATTGTGCAAGA |  | F |
| IGHV1-20*02 | TATTGTGCAAGA |  | F |
| IGHV1-21*01 | TACTGTGCAAGA | CAAGA | P |
| IGHV1-21-1*01 | TACTCTGCAAGA |  | P |
| IGHV1-22*01 | TACTGTGCAAGA | CAAGA | F |
| IGHV1-23*01 | TACTGTACAAGA |  | ORF |
| IGHV1-24*01 | TACTGTGCAAGA | CAAGA | ORF |
| IGHV1-25*01 | TACTGTGCAAGA | CAAGA | P |
| IGHV1-26*01 | TACTGTGCAAGA | CAAGA | F |
| IGHV1-27*01 | TTCTGTGCAAGA |  | P |
| IGHV1-28*01 | TACTTTGCAAGA |  | P |
| IGHV1-31*01 | TACTGTGCAAGA | CAAGA | F |
| IGHV1-32*01 | TACTGTGTAAGA | TAAGA | P |
| IGHV1-34*01 | TACTGTGCAAGA | CAAGA | F |
| IGHV1-34*02 | TACTGTGCAAGA | CAAGA | F |
| IGHV1-35*01 | TACCGTGCAAGACT |  | P |
| IGHV1-35*02 | TACCGTGCAAGA |  | P |
| IGHV1-35*03 |  |  | P |
| IGHV1-36*01 | TACTGTGCAAGA | CAAGA | F |
| IGHV1-37*01 | TATTGTGCAAGA |  | F |
| IGHV1-39*01 | TACTGTGCAAGA | CAAGA | F |
| IGHV1-42*01 | TACTGTGCAAGA | CAAGA | F |
| IGHV1-42*02 |  |  | [F] |
| IGHV1-42*03 |  |  | [P] |
| IGHV1-43*01 | TACTGTGCAAGA | CAAGA | F |
| IGHV1-46*01 | TACTGTGCATGG | CATGG | P |
| IGHV1-47*01 | TACTGTGCAAGG | CAAGG | F |
| IGHV1-48*01 | TATTGTGTAAGA |  | P |
| IGHV1-49*01 | TACTGTGCAAGA | CAAGA | F |
| IGHV1-50*01 | TACTGTGCAAGA | CAAGA | F |
| IGHV1-51*01 | TGTTGTGCTAGACAAGTG |  | P |
| IGHV1-52*01 | TACTGTGCAAGA | CAAGA | F |
| IGHV1-53*01 | TATTGTGCAAGA |  | F |
| IGHV1-53*02 |  |  | [F] |
| IGHV1-53*03 |  |  | [F] |
| IGHV1-53*04 |  |  | [F] |
| IGHV1-54*01 | TTCTGTGCAAGA |  | F |
| IGHV1-54*02 | TTCTGTGCAAGA |  | F |
| IGHV1-54*03 | TTCTGTGCAAGA |  | [F] |
| IGHV1-55*01 | TACTGTGCAAGA | CAAGA | F |
| IGHV1-55*02 |  |  | [F] |
| IGHV1-55*03 |  |  | [F] |
| IGHV1-55*04 |  |  | [F] |
| IGHV1-56*01 | TTCTGTGCAAGA |  | F |
| IGHV1-56*02 | TTCTGTGCAAGA |  | [F] |
| IGHV1-58*01 | TTCTGTGCAAGA |  | F |
| IGHV1-58*02 | TTCTGTGCAAGA |  | P |
| IGHV1-59*01 | TACTGTGCAAGA | CAAGA | F |
| IGHV1-60*01 | TACTGTGCTAGA | CTAGA | P |
| IGHV1-61*01 | TACTGTGCAAGA | CAAGA | F |
| IGHV1-62-1*01 | CTGTGCAAGGAA |  | F |
| IGHV1-62-2*01 | TTCTGTGCAAGACACGAAGA |  | F |
| IGHV1-62-3*01 | TACTGTGCAAGA | CAAGA | ORF |
| IGHV1-62-3*02 |  |  | [F] |
| IGHV1-63*01 | TACTGTGCAAGA | CAAGA | F |
| IGHV1-63*02 | TACTGTGCAAGA | CAAGA | F |
| IGHV1-64*01 | TACTGTGCAAGA | CAAGA | F |
| IGHV1-64*02 |  |  | [F] |
| IGHV1-66*01 | TACTGTGCAAGA | CAAGA | F |
| IGHV1-67*01 | TACTGTGCAAGA | CAAGA | F |
| IGHV1-69*01 | TACTGTGCAAGA | CAAGA | F |
| IGHV1-69*02 | TACTGTGCAAGA | CAAGA | F |
| IGHV1-69*03 |  |  | [F] |
| IGHV1-70*01 | TTCTGTGCAAGA |  | P |
| IGHV1-71*01 | TTCTGTGCAAGACACGAAGA |  | F |
| IGHV1-71*02 | TTCTGTGCAAGACACGAAGA |  | F |
| IGHV1-72*01 | TATTGTGCAAGA |  | F |
| IGHV1-72*02 |  |  | [F] |
| IGHV1-72*03 |  |  | [F] |
| IGHV1-72*04 | TATTGTGCAAGA |  | F |
| IGHV1-72*05 |  |  | [F] |
| IGHV1-74*01 | TACTGTGCAATA | CAATA | F |
| IGHV1-74*02 |  |  | [F] |
| IGHV1-74*03 |  |  | [F] |
| IGHV1-74*04 | TACTGTGCAATA | CAATA | F |
| IGHV1-75*01 | TTCTGTGCAAGA |  | F |
| IGHV1-76*01 | TTCTGTGCAAGA |  | F |
| IGHV1-77*01 | TTCTGTGCAAGA |  | F |
| IGHV1-78*01 | TTCTGTGCAAGA |  | F |
| IGHV1-79*01 | TTCTGTGCAAGA |  | P |
| IGHV1-80*01 | TTCTGTGCAAGA |  | F |
| IGHV1-81*01 | TTCTGTGCAAGA |  | F |
| IGHV1-82*01 | TTCTGTGCAAGA |  | F |
| IGHV1-83*01 | TTCTGTGCAAGA |  | P |
| IGHV1-84*01 | TTCTGTGCAAGA |  | F |
| IGHV1-84*02 | TTCTGTGCAAGA |  | [F] |
| IGHV1-85*01 | TTCTGTGCAAGA |  | F |
| IGHV1S10*01 | TTCTGTGCAAGA |  | P |
| IGHV1S10*02 | TTCTGTGCAAGA |  | P |
| IGHV1S100*01 |  |  | [F] |
| IGHV1S101*01 |  |  | [P] |
| IGHV1S101*02 |  |  | [P] |
| IGHV1S103*01 |  |  | [F] |
| IGHV1S107*01 |  |  | [F] |
| IGHV1S108*01 |  |  | [F] |
| IGHV1S11*01 | TACTGTGCAAGA | CAAGA | P |
| IGHV1S110*01 |  |  | [P] |
| IGHV1S111*01 |  |  | [F] |
| IGHV1S112*01 |  |  | [P] |
| IGHV1S112*02 |  |  | [F] |
| IGHV1S113*01 |  |  | [F] |
| IGHV1S113*02 |  |  | [F] |
| IGHV1S118*01 |  |  | [F] |
| IGHV1S12*01 | TTCTGTGCAAGA |  | F |
| IGHV1S120*01 |  |  | [F] |
| IGHV1S120*02 |  |  | [F] |
| IGHV1S121*01 |  |  | [F] |
| IGHV1S122*01 |  |  | [F] |
| IGHV1S124*01 |  |  | [P] |
| IGHV1S126*01 | TACTGTGCAAGA | CAAGA | [F] |
| IGHV1S127*01 | TACTGTACAAGA |  | [F] |
| IGHV1S130*01 | TACTGTGCAAGA | CAAGA | [F] |
| IGHV1S132*01 | TTCTGTGCAAGA |  | [F] |
| IGHV1S134*01 | TTCTGTGCAAGA |  | [F] |
| IGHV1S135*01 | TACTGTGCAAGA | CAAGA | [F] |
| IGHV1S136*01 | TACTGTGCAAGA | CAAGA | [F] |
| IGHV1S137*01 | TACTGTGCAAGA | CAAGA | [F] |
| IGHV1S14*01 | TACTGTGCAAGA | CAAGA | F |
| IGHV1S15*01 | TATTGTGCAATA |  | P |
| IGHV1S16*01 | TACTGTACAATA |  | P |
| IGHV1S17*01 | TACTGTACAAGA |  | P |
| IGHV1S18*01 | TACTGTGCAAGA | CAAGA | P |
| IGHV1S19*01 | TTCTGTGCAAGA |  | P |
| IGHV1S20*01 | TTCTGTGCAAGA |  | F |
| IGHV1S20*02 | TTCTGTGCAAGA |  | [F] |
| IGHV1S21*01 |  |  | F |
| IGHV1S21*02 |  |  | [F] |
| IGHV1S22*01 | TACTGTACAAGA |  | F |
| IGHV1S26*01 | TACTGTGCAAGA | CAAGA | F |
| IGHV1S28*01 | TACTGTTCGAGA |  | P |
| IGHV1S29*01 |  |  | F |
| IGHV1S29*02 | TACTGTGCAAGA | CAAGA | F |
| IGHV1S30*01 | TACTGTGCAAGA | CAAGA | P |
| IGHV1S31*01 |  |  | F |
| IGHV1S32*01 |  |  | F |
| IGHV1S33*01 |  |  | F |
| IGHV1S34*01 | TACTGTGCAAGA | CAAGA | F |
| IGHV1S35*01 | TACTGTGCAAGA | CAAGA | F |
| IGHV1S36*01 | TACTGTACAAGA |  | F |
| IGHV1S36*02 | TACTGTACAAGA |  | [F] |
| IGHV1S37*01 |  |  | F |
| IGHV1S40*01 | TACTGTGCAAGA | CAAGA | F |
| IGHV1S41*01 | TTCTGTGCAAGA |  | F |
| IGHV1S44*01 |  |  | [F] |
| IGHV1S45*01 | TACTGTGCAAGA | CAAGA | F |
| IGHV1S46*01 | TACTGTGCAAGAT | CAAGAT | [F] |
| IGHV1S47*01 | TTCTGTGCAAGA |  | ORF |
| IGHV1S49*01 | TTCTGCGCAAGA |  | F |
| IGHV1S5*01 | TATTGTGCAAGA |  | F |
| IGHV1S50*01 | TTCTGTGCAAGA |  | F |
| IGHV1S51*01 | TTCTGTGCAAGA |  | P |
| IGHV1S52*01 | TTCTGCGCAAGA |  | F |
| IGHV1S53*01 | TTCTGTAAAAGA |  | F |
| IGHV1S53*02 | TTCTGTAAAAGA |  | F |
| IGHV1S53*03 | TTCTGTAAAAGA |  | F |
| IGHV1S55*01 | TACTGTGCAAGA | CAAGA | F |
| IGHV1S56*01 | TTCTGTGCAAGA |  | F |
| IGHV1S61*01 | TATTGTGCAAGA |  | F |
| IGHV1S65*01 |  |  | [F] |
| IGHV1S65*02 |  |  | [P] |
| IGHV1S65*03 |  |  | [F] |
| IGHV1S67*01 |  |  | [F] |
| IGHV1S67*02 |  |  | [F] |
| IGHV1S68*01 |  |  | [F] |
| IGHV1S68*02 |  |  | [F] |
| IGHV1S70*01 |  |  | [F] |
| IGHV1S72*01 |  |  | [F] |
| IGHV1S73*01 |  |  | [F] |
| IGHV1S74*01 |  |  | [P] |
| IGHV1S75*01 |  |  | [F] |
| IGHV1S75*02 |  |  | [F] |
| IGHV1S78*01 |  |  | [F] |
| IGHV1S81*01 |  |  | [F] |
| IGHV1S81*02 | TACTGTGCAAGA | CAAGA | [F] |
| IGHV1S82*01 |  |  | [F] |
| IGHV1S83*01 |  |  | [F] |
| IGHV1S84*01 |  |  | [P] |
| IGHV1S87*01 |  |  | [F] |
| IGHV1S9*01 | TACTGTGCAAGA | CAAGA | P |
| IGHV1S92*01 |  |  | [F] |
| IGHV1S95*01 |  |  | [F] |
| IGHV1S96*01 |  |  | [F] |
| IGHV10-1*01 | TACTGTGTGAGACA | TGAGACA | F |
| IGHV10-1*02 | TACTGTGTGAGCGA | TGAGCGA | F |
| IGHV10-3*01 | TACTGTGTGAGAG | TGAGAG | F |
| IGHV10-3*02 | TACTGTGTGAGAGA | TGAGAGA | F |
| IGHV10-3*03 | TACTGTGTGAGAGA | TGAGAGA | F |
| IGHV10S3*01 | TACTGTGTGAGAGA | TGAGAGA | F |
| IGHV10S4*01 | TACTGTGTGAGA | TGAGA | F |
| IGHV11-1*01 | TTCTGTATGAGATA |  | F |
| IGHV11-2*01 | TTCTGTATGAGATA |  | F |
| IGHV11-2*02 | TTCTGTATGAGATA |  | F |
| IGHV12-1*01 | TACTGTTCTAGGGAAAACCA |  | P |
| IGHV12-1*02 | TACTATCCCAGGGAAAACCA |  | P |
| IGHV12-1-1*01 | TACTGTTCCAGGGAAAACCA |  | F |
| IGHV12-1-2*01 | TACTGTTCCAGGGAAAACCA |  | P |
| IGHV12-2*01 | TACTATTCCAGGGAAAACCA |  | P |
| IGHV12-2-1*01 | TACTATTCCAGGGAAAACCA |  | ORF |
| IGHV12-3*01 | TACTGTGCAGGAAGACAG | CAGGAAGACAG | F |
| IGHV12-3*02 | TACTGTGCAGGAGACAGA | CAGGAGACAGA | F |
| IGHV13-1*01 | TACTGTGCAAGAGAGA | CAAGAGAGA | F |
| IGHV13-1*02 | TACTGGGCAAGAGAGA |  | ORF |
| IGHV13-2*01 | TTTTGTAGTAGA |  | F |
| IGHV13-2*02 | TATTGTAGTAGA |  | F |
| IGHV13-2*03 | TATTGTAGCAGA |  | (F) |
| IGHV14-1*01 | TACTGTACTACA |  | F |
| IGHV14-1*02 | TACTGTGCTAGA | CTAGA | F |
| IGHV14-2*01 | TACTGTGCTAGA | CTAGA | F |
| IGHV14-2*02 | TACTGTGCTAGA | CTAGA | P |
| IGHV14-3*01 | TACTGTGCTAGA | CTAGA | F |
| IGHV14-3*02 | TACTGTGCTAGA | CTAGA | F |
| IGHV14-4*01 | TACTGTACTACA |  | F |
| IGHV14-4*02 | TACTGTAATGCA |  | F |
| IGHV14S4*01 | TACTGTCCCTAT |  | (F) |
| IGHV15-2*01 | TACTGTGCAAGG | CAAGG | F |
| IGHV15-2*02 | TACTGTGCAAGG | CAAGG | (F) |
| IGHV16-1*01 | TATTGTGCCAGAGA |  | F |
| IGHV2-2*01 | TACTGTGCCAGAAA | CCAGAAA | F |
| IGHV2-2*02 | TACTGTGCCAGAAA | CCAGAAA | F |
| IGHV2-2*03 | TACTGTGCCAGAAA | CCAGAAA | F |
| IGHV2-2-1*01 | TACTGTGCCAGAAA | CCAGAAA | P |
| IGHV2-2-2*01 | TACTGTGTCAGAAA | TCAGAAA | F |
| IGHV2-3*01 | TACTGTGCCAAACC | CCAAACC | F |
| IGHV2-3-1*01 | TACTGTGCCAGAAA | CCAGAAA | F |
| IGHV2-4*01 | TACTGTGCCAAAAA | CCAAAAA | F |
| IGHV2-4*02 | TACTGTGCCAGAAA | CCAGAAA | F |
| IGHV2-4-1*01 | TACTGTGCCAGAAA | CCAGAAA | F |
| IGHV2-5*01 | TACTGTGCCAAAAA | CCAAAAA | F |
| IGHV2-5-1*01 | TACTGTGCCAAAAA | CCAAAAA | F |
| IGHV2-6*01 | TACTGTGCCAGTG | CCAGTG | F |
| IGHV2-6*02 | TACTGTGCCAGAAA | CCAGAAA | F |
| IGHV2-6*03 | TACTGTGCCAGA | CCAGA | F |
| IGHV2-6-1*01 | TACTGTGCCAGACA | CCAGACA | F |
| IGHV2-6-2*01 | TACTGTGCCAGACA | CCAGACA | F |
| IGHV2-6-3*01 | TACTGTGTAAGAGA | TAAGAGA | F |
| IGHV2-6-4*01 | TACTGTGCCAGAAA | CCAGAAA | F |
| IGHV2-6-5*01 | TACTGTGCCAAACA | CCAAACA | F |
| IGHV2-6-6*01 | TACTGTGCCAAACC | CCAAACC | F |
| IGHV2-6-7*01 | TACTGTGCCAGAGA | CCAGAGA | F |
| IGHV2-6-7*02 | TACTGTGCCAGAGA | CCAGAGA | F |
| IGHV2-6-8*01 | TACTGTGCCAGTGA | CCAGTGA | F |
| IGHV2-7*01 | CACTGTGCCAGATA |  | F |
| IGHV2-9*01 | TACTGTGCACAAAC | CACAAAC | F |
| IGHV2-9*02 | TACTGTGTAAGAGA | TAAGAGA | F |
| IGHV2-9-1*01 | TACTGTGCCAGAGA | CCAGAGA | F |
| IGHV2S3*01 | TACTGTGTAAGAGA | TAAGAGA | F |
| IGHV3-1*01 | TACTGTGCAAGAAGGA | CAAGAAGGA | F |
| IGHV3-1*02 | TACTGTGCAAGA | CAAGA | F |
| IGHV3-2*02 | TACTGTGCAAGA | CAAGA | F |
| IGHV3-3*01 | TACTGTGCGAGAGA | CGAGAGA | F |
| IGHV3-3*02 | TACTGTGCGAGAGA | CGAGAGA | P |
| IGHV3-3*03 | TACTGTGCGAGAGA | CGAGAGA | F |
| IGHV3-4*01 | TACTGTGCAAGAGA | CAAGAGA | F |
| IGHV3-4*02 | TACTGTGCAAGAGA | CAAGAGA | F |
| IGHV3-5*01 | TACTGTGCACGAGA | CACGAGA | F |
| IGHV3-5*02 | TACTGTGCACGAGA | CACGAGA | F |
| IGHV3-6*01 | TACTGTGCAAGAGA | CAAGAGA | F |
| IGHV3-6*02 | TACTGTGCAAGAGA | CAAGAGA | F |
| IGHV3-6*03 | TACTGTGCACGA | CACGA | (F) |
| IGHV3-7*01 | TACTGTGCAAACAG | CAAACAG | P |
| IGHV3-7*02 | TACTGTGCAAATGA | CAAATGA | P |
| IGHV3-8*01 | TACTGTGCAAGAAT | CAAGAAT | F |
| IGHV3-8*02 | TACTGTGCAAGATA | CAAGATA | F |
| IGHV3S1*01 | TACTGTGCAAGATA | CAAGATA | F |
| IGHV3S1*02 | TACTGTGCAAGATA | CAAGATA | F |
| IGHV3S7*01 | TACTGTGCAAGAGG | CAAGAGG | (F) |
| IGHV4-1*01 | TACTGTGCAAGA | CAAGA | F |
| IGHV4-1*02 | TACTGTGCAAGACC | CAAGACC | F |
| IGHV4-2*01 | TACTGTGCAAGACC | CAAGACC | P |
| IGHV4-2*02 | TACTGTGCAAGACT | CAAGACT | F |
| IGHV5-1*01 | TACTGTTTGAGAAC |  | P |
| IGHV5-1*02 | TACTGTTTGAGACA |  | P |
| IGHV5-12*01 | TACTGTGCAAGAAC | CAAGAAC | F |
| IGHV5-12*02 | TACTGTGCAAGACA | CAAGACA | F |
| IGHV5-12*03 | TACTGTGCAAGACGA | CAAGACGA | [F] |
| IGHV5-12-1*01 | TACTGTGCAAGACA | CAAGACA | F |
| IGHV5-12-2*01 | TACTGTGCAAGACA | CAAGACA | F |
| IGHV5-12-2*02 | TACTGTGCAAGACA | CAAGACA | F |
| IGHV5-12-4*01 | TACTGTGCAAGA | CAAGA | F |
| IGHV5-15*01 | TACTGTGCAAGAAC | CAAGAAC | F |
| IGHV5-15*02 | TACTGTGCAAGGGA | CAAGGGA | F |
| IGHV5-15*03 | TACTGTGCAAGA | CAAGA | [F] |
| IGHV5-15*04 | TACTGTGCAAGACGA | CAAGACGA | [F] |
| IGHV5-15*05 | TACTGTGCAAGACA | CAAGACA | F |
| IGHV5-16*01 | TACTGTGCAAGAAG | CAAGAAG | F |
| IGHV5-16*02 | TACTGTGCAAGACGA | CAAGACGA | [F] |
| IGHV5-17*01 | TACTGTGCAAGG | CAAGG | F |
| IGHV5-17*02 | TACTGTGCAAGA | CAAGA | F |
| IGHV5-17*03 | TACTGTGCAAGACGA | CAAGACGA | [F] |
| IGHV5-2*01 | TACTGTGCAAGAAC | CAAGAAC | F |
| IGHV5-2*02 | TACTGTGCAAGACA | CAAGACA | F |
| IGHV5-2*03 | TACTGTGCAAGACGA | CAAGACGA | [F] |
| IGHV5-21*01 | TACTGTGCAAGAG | CAAGAG | P |
| IGHV5-4*01 | TACTGTGCAAGAAG | CAAGAAG | F |
| IGHV5-4*02 | TACTGTGCAAGAGA | CAAGAGA | F |
| IGHV5-4*03 | TACTGTGCAAGACGA | CAAGACGA | [F] |
| IGHV5-6*01 | TACTGTGCAAGACA | CAAGACA | F |
| IGHV5-6*02 | TACTGTGCAAGACGA | CAAGACGA | [F] |
| IGHV5-6*03 | TACTGTGCAAGACGA | CAAGACGA | [F] |
| IGHV5-6-1*01 | TACTGTGCAAGACA | CAAGACA | F |
| IGHV5-6-2*01 | TACTGTGCAAGACA | CAAGACA | F |
| IGHV5-6-2*02 | TACTGTGCAAGA | CAAGA | F |
| IGHV5-6-3*01 | TACTGTGCAAGAGA | CAAGAGA | F |
| IGHV5-6-3*02 | TACTGTGCAAGAGA | CAAGAGA | F |
| IGHV5-6-4*01 | TACTGTACAAGAGA |  | F |
| IGHV5-6-4*02 | TACTGTACAAGA |  | F |
| IGHV5-6-5*01 |  |  | F |
| IGHV5-6-6*01 | TACTGTGCAAGA | CAAGA | F |
| IGHV5-9*01 | TACTGTGCAAGAAC | CAAGAAC | F |
| IGHV5-9*02 | TACTGTGCAAGACA | CAAGACA | F |
| IGHV5-9*03 | TACTGTGCAAGATA | CAAGATA | F |
| IGHV5-9*04 | TACTGTGCAAGAC | CAAGAC | [F] |
| IGHV5-9-1*01 | TACTGTGCAAGA | CAAGA | F |
| IGHV5-9-1*02 | TACTGTACAAGA |  | F |
| IGHV5-9-2*01 | TACTGTGCAAGACA | CAAGACA | F |
| IGHV5-9-3*01 | TACTGTGCAAGACA | CAAGACA | F |
| IGHV5-9-4*01 | TACTGTGCAAGGGA | CAAGGGA | F |
| IGHV5-9-5*01 | TACTGTGCAAGATA | CAAGATA | F |
| IGHV5S12*01 | TACTGTGCAAGA | CAAGA | [F] |
| IGHV5S21*01 | TACTGTGCAAGACGA | CAAGACGA | [F] |
| IGHV5S24*01 | TACTGTGCAAGACGA | CAAGACGA | [F] |
| IGHV5S4*01 | TACTGTGCAAGA | CAAGA | F |
| IGHV5S4*02 | TACTGTGCAAGACA | CAAGACA | F |
| IGHV5S9*01 | TACTGTGCAAGACA | CAAGACA | F |
| IGHV6-3*01 | TACTGCACAGG |  | F |
| IGHV6-3*02 | TACTGCACAGG |  | F |
| IGHV6-3*03 | TACTGCACAGGC |  | F |
| IGHV6-4*01 | TACTGTACAAGG |  | F |
| IGHV6-4*02 | TACTGTACAAAGG |  | F |
| IGHV6-5*01 | TACTGTACAAGG |  | F |
| IGHV6-5*02 | TACTGTAAAGG |  | F |
| IGHV6-6*01 | TACTGTACCAGG |  | F |
| IGHV6-6*02 | TACTGTACCAGG |  | F |
| IGHV6-7*01 | TACTGTACATGG |  | F |
| IGHV6-7*02 | TACTGTACATGG |  | F |
| IGHV6S2*01 |  |  | [F] |
| IGHV6S3*01 |  |  | [F] |
| IGHV6S4*01 |  |  | [F] |
| IGHV7-1*01 | TACTGTGCAAGAACGTAG | CAAGAACGTAG | P |
| IGHV7-1*02 | TACTGTGCAAGAGATGCA | CAAGAGATGCA | F |
| IGHV7-1*03 | TACTGTGCAAGAGATGCA | CAAGAGATGCA | F |
| IGHV7-2*01 | TACTGTGCAAGAGATACA | CAAGAGATACA | F |
| IGHV7-3*01 | TACTGTGCAAGATATA | CAAGATATA | F |
| IGHV7-3*02 | TACTGTGCAAGAGATA | CAAGAGATA | F |
| IGHV7-3*03 | TACTGTGCAAAAGATA | CAAAAGATA | F |
| IGHV7-3*04 | TACTGTGCAAGAGATA | CAAGAGATA | F |
| IGHV7-4*01 | TACTGTGTAAAAGCTGTA | TAAAAGCTGTA | F |
| IGHV7-4*02 | TACTGTGCAAAAGATGTA | CAAAAGATGTA | F |
| IGHV7-4*03 | TACTGTGCAAAA | CAAAA | F |
| IGHV7-4*04 | TACTGTGTAAAAGCTGTA | TAAAAGCTGTA | F |
| IGHV8-2*01 | TACGGTGCTTGGAGAGAG |  | ORF |
| IGHV8-4*01 | TACTGTGCT | CT | F |
| IGHV8-5*01 | TACTGTGCTCAAATAG | CTCAAATAG | F |
| IGHV8-5*02 |  |  | [F] |
| IGHV8-6*01 | TACTGTGCTCGA | CTCGA | F |
| IGHV8-7*01 | TACTGTGCT | CT | P |
| IGHV8-8*01 | TACTGTGCTCGAATA | CTCGAATA | F |
| IGHV8-8*02 |  |  | [F] |
| IGHV8-8-1*01 | TACTGTGCTCACAGAC | CTCACAGAC | ORF |
| IGHV8-9*01 | TACTCTGCTCGAAGAG |  | ORF |
| IGHV8-9*02 | TGA |  | [P] |
| IGHV8-9*03 |  |  | [F] |
| IGHV8-10*01 | TACTGTGCTTGAGGAG | CTTGAGGAG | P |
| IGHV8-11*01 | TACTGTGCTCGAATAG | CTCGAATAG | F |
| IGHV8-12*01 | TACTGTGCTCGAAGAG | CTCGAAGAG | F |
| IGHV8-13*01 | TACTGTGCTCGAAG | CTCGAAG | ORF |
| IGHV8-14*01 | TACTGTGCTTGAGGAG | CTTGAGGAG | P |
| IGHV8S2*01 | TACTGTGCTTGAGGAG | CTTGAGGAG | P |
| IGHV8S6*01 |  |  | [P] |
| IGHV8S9*01 |  |  | [F] |
| IGHV9-1*01 | TTCTGTGTAAGA |  | F |
| IGHV9-1*02 | TTCTGTGCAAGA |  | F |
| IGHV9-1*03 |  |  | F |
| IGHV9-1*04 |  |  | F |
| IGHV9-2*01 | TTCTGTGCAAGA |  | F |
| IGHV9-2*02 | TTCTGTGCAAGA |  | F |
| IGHV9-2-1*01 | TTCTGTGCTAGA |  | F |
| IGHV9-3*01 | TTCTGTGCAAGA |  | F |
| IGHV9-3*02 | TTCTGTGCAAGA |  | F |
| IGHV9-3*03 | TTCTGT |  | F |
| IGHV9-3-1*01 | TTCTGTGCAAGA |  | F |
| IGHV9-4*01 | TTCTGTGCGAGAA |  | F |
| IGHV9-4*02 | TTCTGTGCGAGA |  | F |
| IGHV9-4*03 | TTCTGT |  | F |
| IGHV9S7*01 | TTCTGT |  | F |
| IGHV9S8*01 | TTCTGT |  | F |
