# Supplementary material for: Contribution of VH Replacement Products in Mouse Antibody Repertoire
Source: PLoS One. 2013 Feb 28;8(2):e57877. doi: 10.1371/journal.pone.0057877 (PMC3585286; doi:10.1371/journal.pone.0057877)
Supplement: Table S4 — Potential mouse VH replacement footprint motifs with different length. (DOCX) [file pone.0057877.s004.docx]

| **Table S4. Potential mouse V_H_ replacement footprint motifs with different length.** | | | | | | | | |  |
| --- | --- | --- | --- | --- | --- | --- | --- | --- | --- |
| **length(#)** | **11(6)** | **10(12)** | **9(25)** | **8(36)** | **7(68)** | **6(85)** | **5(84)** | **4(72)** | **3(42)** |
| **potential  footprints** | CAGGAAGACAG | CAGGAAGACA | CAGGAAGAC | CAGGAAGA | TGAGACA | TGAGAC | TGAGA | TGAG | TGA |
|  | CAGGAGACAGA | AGGAAGACAG | AGGAAGACA | AGGAAGAC | TGAGCGA | GAGACA | GAGAC | GAGA | GAG |
|  | CAAGAGATGCA | CAGGAGACAG | GGAAGACAG | GGAAGACA | TGAGAGA | TGAGCG | AGACA | AGAC | AGA |
|  | CAAGAGATACA | AGGAGACAGA | CAGGAGACA | GAAGACAG | GAAGAGG | GAGCGA | TGAGC | GACA | GAC |
|  | TAAAAGCTGTA | CAAGAGATGC | AGGAGACAG | CAGGAGAC | CAGGAAG | TGAGAG | GAGCG | GAGC | ACA |
|  | CAAAAGATGTA | AAGAGATGCA | GGAGACAGA | AGGAGACA | AGGAAGA | GAGAGA | AGCGA | AGCG | AGC |
|  |  | CAAGAGATAC | CAAGAGAGA | GGAGACAG | GGAAGAC | GAAGAG | GAGAG | GCGA | GCG |
|  |  | AAGAGATACA | CAAGAAGGA | GAGACAGA | GAAGACA | AAGAGG | AGAGA | AGAG | CGA |
|  |  | TAAAAGCTGT | CAAGAGATG | CAAGAGAG | AAGACAG | CAGGAA | GAAGA | GAAG | GAA |
|  |  | AAAAGCTGTA | AAGAGATGC | AAGAGAGA | CAGGAGA | AGGAAG | AAGAG | AAGA | AAG |
|  |  | CAAAAGATGT | AGAGATGCA | CAAGAAGG | AGGAGAC | GGAAGA | AGAGG | GAGG | AGG |
|  |  | AAAAGATGTA | CAAGAGATA | AAGAAGGA | GGAGACA | GAAGAC | CAAGA | CAAG | CAA |
|  |  |  | AAGAGATAC | CAAGAGAT | GAGACAG | AAGACA | CAGGA | CAGG | CAG |
|  |  |  | AGAGATACA | AAGAGATG | AGACAGA | AGACAG | AGGAA | AGGA | GGA |
|  |  |  | CAAGATATA | AGAGATGC | CAAGAGA | CAGGAG | GGAAG | GGAA | CTA |
|  |  |  | CAAAAGATA | GAGATGCA | AAGAGAG | AGGAGA | AAGAC | ACAG | TAG |
|  |  |  | TAAAAGCTG | AAGAGATA | AGAGAGA | GGAGAC | GACAG | GGAG | AAT |
|  |  |  | AAAAGCTGT | AGAGATAC | CCAGAAA | GACAGA | AGGAG | CAGA | ATA |
|  |  |  | AAAGCTGTA | GAGATACA | TCAGAAA | CAAGAG | GGAGA | CTAG | CCA |
|  |  |  | CAAAAGATG | CAAGATAT | CCAAACC | AAGAGA | ACAGA | TAGA | AAA |
|  |  |  | AAAAGATGT | AAGATATA | CCAAAAA | AGAGAG | CTAGA | AAGG | TCA |
|  |  |  | AAAGATGTA | CAAAAGAT | CCAGACA | CCAGAA | CAAGG | CAAT | AAC |
|  |  |  | CTCGAATAG | AAAAGATA | TAAGAGA | CAGAAA | CAATA | AATA | ACC |
|  |  |  | CTCGAAGAG | TAAAAGCT | CCAAACA | TCAGAA | CCAGA | CCAG | AGT |
|  |  |  | CTCAAATAG | AAAAGCTG | CCAGAGA | CCAAAC | CAGAA | AGAA | GTG |
|  |  |  |  | AAAGCTGT | CCAGTGA | CAAACC | AGAAA | GAAA | TAA |
|  |  |  |  | AAGCTGTA | CACAAAC | CCAAAA | TCAGA | TCAG | CAC |
|  |  |  |  | AAAAGATG | CAAGAAG | CAAAAA | CCAAA | CCAA | ACG |
|  |  |  |  | AAAGATGT | AAGAAGG | CCAGTG | CAAAC | CAAA | GAT |
|  |  |  |  | AAGATGTA | AGAAGGA | CCAGAC | AAACC | AAAC | ACT |
|  |  |  |  | CTCGAATA | CGAGAGA | CAGACA | CAAAA | AACC | ATG |
|  |  |  |  | TCGAATAG | CACGAGA | TAAGAG | AAAAA | AAAA | TGC |
|  |  |  |  | CTCGAAGA | CAAGAAT | CAAACA | CCAGT | CAGT | GCA |
|  |  |  |  | TCGAAGAG | CAAGATA | CCAGAG | CAGTG | AGTG | TAC |
|  |  |  |  | CTCAAATA | CAAGACC | CAGAGA | CAGAC | TAAG | TAT |
|  |  |  |  | TCAAATAG | CAAGACT | CAGTGA | TAAGA | AACA | GCT |
|  |  |  |  |  | CAAGAAC | CACAAA | AAACA | GTGA | CTG |
|  |  |  |  |  | CAAGACA | ACAAAC | CAGAG | CACA | TGT |
|  |  |  |  |  | AAGAGAT | CAAGAA | AGTGA | ACAA | GTA |
|  |  |  |  |  | AGAGATG | AAGAAG | CACAA | CGAG | CTC |
|  |  |  |  |  | GAGATGC | AGAAGG | ACAAA | CACG | TCG |
|  |  |  |  |  | AGATGCA | GAAGGA | AAGAA | ACGA |  |
|  |  |  |  |  | AGAGATA | CGAGAG | AGAAG | GAAT |  |
|  |  |  |  |  | GAGATAC | CACGAG | GAAGG | AGAT |  |
|  |  |  |  |  | AGATACA | ACGAGA | AAGGA | GATA |  |
|  |  |  |  |  | AAGATAT | AAGAAT | CGAGA | GACC |  |
|  |  |  |  |  | AGATATA | CAAGAT | CACGA | GACT |  |
|  |  |  |  |  | CAAAAGA | AAGATA | ACGAG | GAAC |  |
|  |  |  |  |  | AAAAGAT | CAAGAC | AGAAT | GATG |  |
|  |  |  |  |  | AAAGATA | AAGACC | AAGAT | ATGC |  |
|  |  |  |  |  | TAAAAGC | AAGACT | AGATA | TGCA |  |
|  |  |  |  |  | AAAAGCT | AAGAAC | AGACC | ATAC |  |
|  |  |  |  |  | AAAGCTG | AGAGAT | AGACT | TACA |  |
|  |  |  |  |  | AAGCTGT | GAGATG | AGAAC | ATAT |  |
|  |  |  |  |  | AGCTGTA | AGATGC | GAGAT | TATA |  |
|  |  |  |  |  | AAAGATG | GATGCA | AGATG | AAAG |  |
|  |  |  |  |  | AAGATGT | GAGATA | GATGC | TAAA |  |
|  |  |  |  |  | AGATGTA | AGATAC | ATGCA | AAGC |  |
|  |  |  |  |  | CTCGAAT | GATACA | GATAC | AGCT |  |
|  |  |  |  |  | TCGAATA | AGATAT | ATACA | GCTG |  |
|  |  |  |  |  | CGAATAG | GATATA | GATAT | CTGT |  |
|  |  |  |  |  | CTCGAAG | CAAAAG | ATATA | TGTA |  |
|  |  |  |  |  | TCGAAGA | AAAAGA | AAAAG | ATGT |  |
|  |  |  |  |  | CGAAGAG | AAAGAT | AAAGA | CTCG |  |
|  |  |  |  |  | CTCAAAT | TAAAAG | TAAAA | TCGA |  |
|  |  |  |  |  | TCAAATA | AAAAGC | AAAGC | CGAA |  |
|  |  |  |  |  | CAAATAG | AAAGCT | AAGCT | ATAG |  |
|  |  |  |  |  |  | AAGCTG | AGCTG | CTCA |  |
|  |  |  |  |  |  | AGCTGT | GCTGT | TCAA |  |
|  |  |  |  |  |  | GCTGTA | CTGTA | AAAT |  |
|  |  |  |  |  |  | AAGATG | GATGT |  |  |
|  |  |  |  |  |  | AGATGT | ATGTA |  |  |
|  |  |  |  |  |  | GATGTA | CTCGA |  |  |
|  |  |  |  |  |  | CTCGAA | TCGAA |  |  |
|  |  |  |  |  |  | TCGAAT | CGAAT |  |  |
|  |  |  |  |  |  | CGAATA | GAATA |  |  |
|  |  |  |  |  |  | GAATAG | AATAG |  |  |
|  |  |  |  |  |  | TCGAAG | CGAAG |  |  |
|  |  |  |  |  |  | CGAAGA | CTCAA |  |  |
|  |  |  |  |  |  | CTCAAA | TCAAA |  |  |
|  |  |  |  |  |  | TCAAAT | CAAAT |  |  |
|  |  |  |  |  |  | CAAATA | AAATA |  |  |
|  |  |  |  |  |  | AAATAG |  |  |  |
