# Supplementary material for: Contribution of VH Replacement Products in Mouse Antibody Repertoire
Source: PLoS One. 2013 Feb 28;8(2):e57877. doi: 10.1371/journal.pone.0057877 (PMC3585286; doi:10.1371/journal.pone.0057877)
Supplement: Table S5 — Identification of 4-mer VH replacement footprint motifs in mouse IgH sequences. (DOCX) [file pone.0057877.s005.docx]

| **Table S5. Identification of 4-mer V_H_ replacement footprint motifs in mouse IgH sequences.** | | | | | | | |
| --- | --- | --- | --- | --- | --- | --- | --- |
| **Accession #** | **V_H_ Gene** | **V_H_** | **P** | **N1** | **P** | **D_H_** | **CDR3 (AA)** |
| FJ816454.1 | IGHV1-26*01 | tgtgcaaga |  | gattgggaa |  | actacggc | ARDWETTAFAY |
| GU906999.1 | IGHV14-3*02 | tgtgctaga |  | ggagga |  | tatggt | ARGGYGPFAY |
| GU906999.1 | IGHV14-3*02 | tgtgctaga |  | ggagga |  | tatggt | ARGGYGPFAY |
| GU906999.1 | IGHV14-3*02 | tgtgctaga |  | ggagga |  | tatggt | ARGGYGPFAY |
| FJ816315.1 | IGHV1-76*01 | tgtgcaa |  | agggggag |  | actacggctac | AKGETTATGAMGY |
| FJ816321.1 | IGHV1-76*01 | tgtgcaa |  | agggggag |  | actacggctac | AKGETTATGAMDY |
| FJ816511.1 | IGHV1-9*01 | tgtgcaag |  | gagggg |  | tattactacggtagta | ARRGITTVVKYYFDY |
| FJ816324.1 | IGHV1-76*01 | tgtgcaa |  | agggggag |  | actacggctac | AKGETTATGAMDY |
| FJ816482.1 | IGHV3-5*02 | tgtgcacggga | t | ggagc |  | ttacgacgg | ARDGAYDGYFDY |
| FJ816482.1 | IGHV3-5*02 | tgtgcacggga | t | ggagc |  | ttacgacgg | ARDGAYDGYFDY |
| FJ816459.1 | IGHV14-3*02 | tgtgctaga |  | atgaccct |  | tacggtagtagctac | ARMTLTVVATGNY |
| FJ816560.1 | IGHV14-3*02 | tgtgctaga |  | ggaggg |  | tattctc | ARGGYSRYYAMDY |
| FJ816560.1 | IGHV14-3*02 | tgtgctaga |  | ggaggg |  | tattctc | ARGGYSRYYAMDY |
| FJ816323.1 | IGHV1-76*01 | tgtgcaa |  | agggggag |  | actacggctac | AKGETTATGAMDY |
| FJ816550.1 | IGHV1-4*01 | tgtgcaaga |  | gaaa |  | agtatggtaact | AREKYGNFFAY |
| FJ816363.1 | IGHV1-14*01 | tgtgcaaga |  | ggag |  | attactacggtagtagc | ARGDYYGSSPFAY |
| FJ816303.1 | IGHV1S132*01 | tgtgcaa |  | agggggag |  | actacggct | AKGETTAAGAMDY |
| FJ816302.1 | IGHV1S132*01 | tgtgcaa |  | agggggag |  | actacggctac | AKGETTATGAMDY |
| GU906996.1 | IGHV14-3*02 | tgtgctaga |  | ggagga |  | tatggt | ARGGYGPFAY |
| GU906996.1 | IGHV14-3*02 | tgtgctaga |  | ggagga |  | tatggt | ARGGYGPFAY |
| GU906996.1 | IGHV14-3*02 | tgtgctaga |  | ggagga |  | tatggt | ARGGYGPFAY |
| GU907040.1 | IGHV1-4*01 | tgtgcaaga | tc | gaagg |  | gatggtta | ARSKGWLVFAY |
| GU906998.1 | IGHV14-3*02 | tgtgctaga |  | ggagga |  | tatggt | ARGGYGPFAY |
| GU906998.1 | IGHV14-3*02 | tgtgctaga |  | ggagga |  | tatggt | ARGGYGPFAY |
| GU906998.1 | IGHV14-3*02 | tgtgctaga |  | ggagga |  | tatggt | ARGGYGPFAY |
| FJ816304.1 | IGHV1-76*01 | tgtgcaa |  | agggggag |  | actacggctac | AKGETTATGAMDY |
| FJ816293.1 | IGHV1-9*01 | tgtgcaa |  | agggggag |  | actacggctac | AKGETTATGAMDY |
| GU906995.1 | IGHV14-3*02 | tgtgctgga |  | ggagga |  | tatggt | AGGGYGPFAY |
| GU906995.1 | IGHV14-3*02 | tgtgctgga |  | ggagga |  | tatggt | AGGGYGPFAY |
| GU906995.1 | IGHV14-3*02 | tgtgctgga |  | ggagga |  | tatggt | AGGGYGPFAY |
| FJ816442.1 | IGHV14-1*02 | tgtgcta |  | aaacctc |  | cttcggtagtagct | AKTSFGSSSWYFDV |
| GU906997.1 | IGHV14-3*02 | tgtgctaga |  | ggagga |  | tatggt | ARGGYGPFAY |
| GU906997.1 | IGHV14-3*02 | tgtgctaga |  | ggagga |  | tatggt | ARGGYGPFAY |
| GU906997.1 | IGHV14-3*02 | tgtgctaga |  | ggagga |  | tatggt | ARGGYGPFAY |
| GU907020.1 | IGHV1-74*01 | tgtgcaa |  | gaggg |  | attactacggtagtagc | ARGITTVVAHFDY |
| FJ816298.1 | IGHV1S22*01 | tgtgcaa |  | agggggag |  | actacggctac | AKGETTATGAMDY |
| GU906994.1 | IGHV14-3*02 | tgtgctaga |  | ggagga |  | tatggt | ARGGYGPFAY |
| GU906994.1 | IGHV14-3*02 | tgtgctaga |  | ggagga |  | tatggt | ARGGYGPFAY |
| GU906994.1 | IGHV14-3*02 | tgtgctaga |  | ggagga |  | tatggt | ARGGYGPFAY |
| GU907012.1 | IGHV1-63*02 | tgtgcaag |  | gttccaa |  | tactacggtagtagttac | ARFQYYGSSYLYYFDF |
| GU906993.1 | IGHV14-3*02 | tgtgctaga |  | ggagga |  | tatggt | ARGGYGPFAY |
| GU906993.1 | IGHV14-3*02 | tgtgctaga |  | ggagga |  | tatggt | ARGGYGPFAY |
| GU906993.1 | IGHV14-3*02 | tgtgctaga |  | ggagga |  | tatggt | ARGGYGPFAY |
| GU906989.1 | IGHV14-3*02 | tgtgctaga |  | ggagga |  | tatggt | ARGGYGPFAY |
| GU906989.1 | IGHV14-3*02 | tgtgctaga |  | ggagga |  | tatggt | ARGGYGPFAY |
| GU906989.1 | IGHV14-3*02 | tgtgctaga |  | ggagga |  | tatggt | ARGGYGPFAY |
| GU906990.1 | IGHV14-3*02 | tgtgctaga |  | ggagga |  | tatggt | ARGGYGPFAY |
| GU906990.1 | IGHV14-3*02 | tgtgctaga |  | ggagga |  | tatggt | ARGGYGPFAY |
| GU906990.1 | IGHV14-3*02 | tgtgctaga |  | ggagga |  | tatggt | ARGGYGPFAY |
| FJ816296.1 | IGHV1-76*01 | tgtgcaa |  | agggggag |  | actacggctac | AKGETTATGAMDY |
| FJ816300.1 | IGHV1-76*01 | tgtgcaa |  | agggggag |  | actacggctac | AKGETTATGAMDY |
| FJ816301.1 | IGHV1S132*01 | tgtgcaa |  | agggggag |  | actacggctac | AKGETTATGAMDY |
| FJ816471.1 | IGHV5-9-3*01 | tgtgcaag |  | tggag |  | acggtagtag | ASGDGSSYWYFDV |
| FJ816311.1 | IGHV1-76*01 | tgtgcaa |  | agggggag |  | actacggctac | AKGETTATGAMDY |
| FJ816306.1 | IGHV1-76*01 | tgtgcaa |  | agggggag |  | actacggctac | AKGETTATGAMDY |
| FJ816305.1 | IGHV1-76*01 | tgtgcaa |  | agggggag |  | actacggctac | AKGETTATGAMDY |
| FJ816546.1 | IGHV1-4*01 | tgtgcaag |  | gaagagg |  | acgggag | ARKRTGGNYFDY |
| FJ816474.1 | IGHV2-9-1*01 | tgtgccagaga | tc | gagg | a | tttattactac | ARDRGFITTNYAMDY |
| FJ816309.1 | IGHV1-76*01 | tgcgcaa |  | agggggag |  | actacggctac | AKGETTATGAMDY |
| FJ816609.1 | IGHV14-4*02 | tgtaatgca | t | ttggag |  | gattacg | NAFGGLRGYFDV |
| FJ816299.1 | IGHV1-76*01 | tgtgcaa |  | agggggag |  | actacggctac | AKGETTATGAMDY |
| GU907039.1 | IGHV1-4*01 | tgtgcaaga | tc | gaagg |  | gatggtta | ARSKGWLVFAY |
| FJ816295.1 | IGHV1-63*02 | tgtgcaa |  | agggggag |  | actacggctac | AKGETTATGAMDY |
| FJ816359.1 | IGHV14-3*02 | tgtgc |  | aaagggggag |  | actacggctac | AKGETTATGAMDY |
| FJ816359.1 | IGHV14-3*02 | tgtgc |  | aaagggggag |  | actacggctac | AKGETTATGAMDY |
| FJ816359.1 | IGHV14-3*02 | tgtgc |  | aaagggggag |  | actacggctac | AKGETTATGAMDY |
| FJ816537.1 | IGHV1-74*01 | tgtgcaa |  | gagagg |  | acgac | AREDDVFAY |
| FJ816537.1 | IGHV1-74*01 | tgtgcaa |  | gagagg |  | acgac | AREDDVFAY |
| FJ816452.1 | IGHV14-3*02 | tgtgctag |  | gattgtattgcgggggtcaagc |  | atagtt | ARIVLRGSSIVYYYAMDY |
| FJ816452.1 | IGHV14-3*02 | tgtgctag |  | gattgtattgcgggggtcaagc |  | atagtt | ARIVLRGSSIVYYYAMDY |
| FJ816452.1 | IGHV14-3*02 | tgtgctag |  | gattgtattgcgggggtcaagc |  | atagtt | ARIVLRGSSIVYYYAMDY |
| FJ816452.1 | IGHV14-3*02 | tgtgctag |  | gattgtattgcgggggtcaagc |  | atagtt | ARIVLRGSSIVYYYAMDY |
| FJ816590.1 | IGHV14-3*02 | tgtgctaga |  | gaggg |  | ctatggtaact | AREGYGNSPFDY |
| FJ816328.1 | IGHV2-2*02 | tgtgccagaa |  | gaga | a | tctactatgattacga | ARRESTMITTWFAY |
| GU907010.1 | IGHV1-5*01 | tgtacaaga |  | gagac |  | ctatggtaactac | TRETYGNYAYYFDY |
| GU906991.1 | IGHV14-3*02 | tgtgctaga |  | ggagga |  | tatggt | ARGGYGPFAY |
| GU906991.1 | IGHV14-3*02 | tgtgctaga |  | ggagga |  | tatggt | ARGGYGPFAY |
| GU906991.1 | IGHV14-3*02 | tgtgctaga |  | ggagga |  | tatggt | ARGGYGPFAY |
| GU907014.1 | IGHV1-63*02 | tgtgcaag |  | gttccaa |  | tactacggtagtagttac | ARFQYYGSSYLYYFDF |
| GU906992.1 | IGHV14-3*02 | tgtgctaga |  | ggagga |  | tatggt | ARGGYGPFAY |
| GU906992.1 | IGHV14-3*02 | tgtgctaga |  | ggagga |  | tatggt | ARGGYGPFAY |
| GU906992.1 | IGHV14-3*02 | tgtgctaga |  | ggagga |  | tatggt | ARGGYGPFAY |
| FJ816400.1 | IGHV1-76*01 | tgtgcaag |  | gaac |  | acgggg | ARNTGAMDY |
| FJ816313.1 | IGHV1-76*01 | tgtgcaa |  | agggggag |  | actacggctac | AKGETTATGAMDY |
| FJ816307.1 | IGHV1-76*01 | tgtgcaa |  | agggggag |  | actacggctac | AKGETTATGAMDH |
| FJ816308.1 | IGHV1-76*01 | tgtgcaa |  | agggggag |  | actacggctac | AKGETTATGAMDY |
| GU907018.1 | IGHV1-9*01 | tgtgccaga |  | ggagga |  | tatggt | ARGGYGPFAY |
| GU907018.1 | IGHV1-9*01 | tgtgccaga |  | ggagga |  | tatggt | ARGGYGPFAY |
| GU907018.1 | IGHV1-9*01 | tgtgccaga |  | ggagga |  | tatggt | ARGGYGPFAY |
| GU907030.1 | IGHV14-4*02 | tgt |  | gcaacatcggc |  | gtatggtaactac | ATSAYGNYGFDY |
| FJ816297.1 | IGHV1-84*02 | tgtgcaa |  | agggggag |  | actacggctac | AKGETTATGAMDY |
| FJ816514.1 | IGHV1-55*01 | tgtgcaaga |  | ggaa |  | aactgggac | ARGKLGRLWYFDV |
| GU907013.1 | IGHV1-63*02 | tgtgcaag |  | gttccaa |  | tactacggtagtagttac | ARFQYYGSSYLYYFDF |
| FJ816312.1 | IGHV1-76*01 | tgtgcaa |  | agggggag |  | actacggctac | AKGETTATGAMDY |
| FJ816574.1 | IGHV14-4*02 | tgtaatg |  | gacct | a | tctactatgattacg | NGPIYYDYGYYFDY |
| FJ816576.1 | IGHV1-20*02 | tgtgcaaga |  | aaggggg |  | acggtagtagctac | ARKGDGSSYVAMDY |
| FJ816316.1 | IGHV1-76*01 | tgtgcaa |  | agggggag |  | actacggctac | AKGETTATGAVDY |
| FJ816314.1 | IGHV1-76*01 | tgtgcaa |  | agggggag |  | actacggctac | AKGETTATGAMDY |
| FJ816310.1 | IGHV1-76*01 | tgtgcaa |  | agggggag |  | actacggctac | AKGETTATGAMDY |
| GU907019.1 | IGHV1-74*01 | tgtgcaa |  | gaggg |  | attactacggtagtagc | ARGITTVVAHFDY |
| FJ816495.1 | IGHV1-47*01 | tgtgcaagg |  | gagag |  | ttactacggt | ARESYYGPFAY |
| FJ816317.1 | IGHV1-76*01 | tgtgcaa |  | agggggag |  | acttcggctac | AKGETSATGAMDY |
| FJ816319.1 | IGHV1-76*01 | tgtgcaa |  | agggggag |  | actacggctac | AKGETTATGAMDY |
| FJ816320.1 | IGHV1-76*01 | tgtgcaa |  | agggggag |  | actacggctac | AKGETTATGAMDY |
| GU907038.1 | IGHV1-4*01 | tgtgcaaga | tc | gaagg |  | gatggtta | ARSKGWLVFAY |
| GU907009.1 | IGHV14-4*02 | tgt |  | gctcagtc |  | tgatggttactac | AQSDGYYAWFAY |
| GU907009.1 | IGHV14-4*02 | tgt |  | gctcagtc |  | tgatggttactac | AQSDGYYAWFAY |
| GU907009.1 | IGHV14-4*02 | tgt |  | gctcagtc |  | tgatggttactac | AQSDGYYAWFAY |
| FJ816322.1 | IGHV1-76*01 | tgtgcaa |  | agggggag |  | actacggctac | AKGETTATGAMDY |
| GU907008.1 | IGHV14-4*02 | tgt |  | gctagaggagga |  | tatggt | ARGGYGPFAY |
| GU907008.1 | IGHV14-4*02 | tgt |  | gctagaggagga |  | tatggt | ARGGYGPFAY |
| GU907008.1 | IGHV14-4*02 | tgt |  | gctagaggagga |  | tatggt | ARGGYGPFAY |
| GU907008.1 | IGHV14-4*02 | tgt |  | gctagaggagga |  | tatggt | ARGGYGPFAY |
| GU907008.1 | IGHV14-4*02 | tgt |  | gctagaggagga |  | tatggt | ARGGYGPFAY |
| FJ816358.1 | IGHV14-3*02 | tgtgc |  | aaagggggag |  | actacggctac | AKGETTATGAMDY |
| FJ816358.1 | IGHV14-3*02 | tgtgc |  | aaagggggag |  | actacggctac | AKGETTATGAMDY |
| FJ816358.1 | IGHV14-3*02 | tgtgc |  | aaagggggag |  | actacggctac | AKGETTATGAMDY |
| GU907002.1 | IGHV14-3*02 | tgtgctaga |  | ggagga |  | tatggt | ARGGYGPFAY |
| GU907002.1 | IGHV14-3*02 | tgtgctaga |  | ggagga |  | tatggt | ARGGYGPFAY |
| GU907002.1 | IGHV14-3*02 | tgtgctaga |  | ggagga |  | tatggt | ARGGYGPFAY |
| GU907000.1 | IGHV14-3*02 | tgtgctaga |  | ggagga |  | tatggt | ARGGYGPFAY |
| GU907000.1 | IGHV14-3*02 | tgtgctaga |  | ggagga |  | tatggt | ARGGYGPFAY |
| GU907000.1 | IGHV14-3*02 | tgtgctaga |  | ggagga |  | tatggt | ARGGYGPFAY |
| GU907001.1 | IGHV14-3*02 | tgtgctaga |  | ggagga |  | tatggt | ARGGYGPFAY |
| GU907001.1 | IGHV14-3*02 | tgtgctaga |  | ggagga |  | tatggt | ARGGYGPFAY |
| GU907001.1 | IGHV14-3*02 | tgtgctaga |  | ggagga |  | tatggt | ARGGYGPFAY |
| FJ816325.1 | IGHV1-76*01 | tgtgcaa |  | agggggag |  | actacggctac | AKGETTATGAMDY |
| FJ816522.1 | IGHV2-9-1*01 | tgtgccagaga | tc | ggggatatcg |  | tactgggggt | ARDRGYRTGGFDY |
| FJ816520.1 | IGHV1S132*01 | tgtgcaaga |  | gggaggacct |  | cctactataggtacgac | ARGRTSYYRYDVDFDY |
| FJ816520.1 | IGHV1S132*01 | tgtgcaaga |  | gggaggacct |  | cctactataggtacgac | ARGRTSYYRYDVDFDY |
| FJ816520.1 | IGHV1S132*01 | tgtgcaaga |  | gggaggacct |  | cctactataggtacgac | ARGRTSYYRYDVDFDY |
| FJ816520.1 | IGHV1S132*01 | tgtgcaaga |  | gggaggacct |  | cctactataggtacgac | ARGRTSYYRYDVDFDY |
| FJ816456.1 | IGHV1-84*02 | tgtgc |  | cgccag |  | gggtcatcta | AARGHLIFDY |
