# Supplementary material for: Contribution of VH Replacement Products in Mouse Antibody Repertoire
Source: PLoS One. 2013 Feb 28;8(2):e57877. doi: 10.1371/journal.pone.0057877 (PMC3585286; doi:10.1371/journal.pone.0057877)
Supplement: Table S6 — Identification of VH replacement products in IgH genes correlating with different keywords. (DOCX) [file pone.0057877.s006.docx]

| **Table S6. Identification of V_H_ replacement products in IgH genes correlating with different keywords.** | | | | | | | |
| --- | --- | --- | --- | --- | --- | --- | --- |
| **BALB/c** |  |  |  |  |  |  |  |
| **Accession #** | **V_H_ Gene** | **V_H_** | **P** | **N1^a^** | **P** | **D_H_** | **CDR3 (aa)^b^** |
| AY077780.1 | IGHV1-42*01 | tgtg |  | ttagaag |  | tggtaactac | VRSGNYEEYAMDY |
| AF290570.1 | IGHV14-3*02 | tgtgcta |  | tagaga |  | aggatctac | AIEKDLP |
| DQ341293.1 | IGHV1-5*01 | tgtgcaaga |  | ggggaggaaa |  | taggggtacgac | ARGEEIGVRRWFAY |
| AY171993.1 | IGHV3-8*02 | tgtgcaag |  | gggatttgagaa | a | tctatga | ARGFEKSMTLDY |
| AY171944.1 | IGHV1-4*01 | tgtgcaaga | t | ggggaga |  | atcggtacgac | ARWGESVRRGDYAMDY |
| AY816715.1 | IGHV2-9-1*01 | tgtgccagag |  | ggggaagtagg |  | tacggtagtagctac | ARGGSRYGSSYDSMDF |
| U70449.1 | IGHV5-12*02 | tgtgc |  | ggcgagagagggtc |  | tcttctatgattacg | AAREGLFYDYVVYFDY^c^ |
| U70449.1 | IGHV5-12*02 | tgtgc |  | ggcgagagagggtc |  | tcttctatgattacg | AAREGLFYDYVVYFDY |
| U70449.1 | IGHV5-12*02 | tgtgc |  | ggcgagagagggtc |  | tcttctatgattacg | AAREGLFYDYVVYFDY |
| M76418.1 | IGHV2-6-5*01 | tgtg |  | gcgggcatgaaggg |  | tacggtagtag | GGHEGYGSSTWFAY |
| AY877966.1 | IGHV5-12*03 | tgtgcaag |  | gggaga |  | tactacggt | ARGDTTVMDY |
| U01850.1 | IGHV5-9*04 | tgtgcaag |  | ggagagggg |  | ttactacggtagtagct | ARERGYYGSSSYFDY |
| U01850.1 | IGHV5-9*04 | tgtgcaag |  | ggagagggg |  | ttactacggtagtagct | ARERGYYGSSSYFDY |
| U01850.1 | IGHV5-9*04 | tgtgcaag |  | ggagagggg |  | ttactacggtagtagct | ARERGYYGSSSYFDY |
| AY369874.1 | IGHV1-37*01 | tgtggaaga |  | ggagag |  | attactacggtagtagctac | GRGEITTVVATSAMDY |
| AY369874.1 | IGHV1-37*01 | tgtggaaga |  | ggagag |  | attactacggtagtagctac | GRGEITTVVATSAMDY |
| AY172006.1 | IGHV3-2*02 | tgtgcaaga |  | ggaagag |  | attactacggtagtagc | ARGRDYYGSSPYYAMDY |
| AY172006.1 | IGHV3-2*02 | tgtgcaaga |  | ggaagag |  | attactacggtagtagc | ARGRDYYGSSPYYAMDY |
| AY172864.1 | IGHV2-9-1*01 | tgtgccagag |  | gcagaa |  | atggtaact | ARGRNGNYYFDY |
| AY172369.1 | IGHV1S28*01 | tgt |  | gcaagat |  | ggggcagctcgggct | ARWGSSGFYFDY |
| L14733.1 | IGHV1-74*01 | tgtgcaa |  | gaggagaatatggtaa |  | ctacgtttattactata | ARGEYGNYVYYYTMHY |
| L14733.1 | IGHV1-74*01 | tgtgcaa |  | gaggagaatatggtaa |  | ctacgtttattactata | ARGEYGNYVYYYTMHY |
| L14733.1 | IGHV1-74*01 | tgtgcaa |  | gaggagaatatggtaa |  | ctacgtttattactata | ARGEYGNYVYYYTMHY |
| GQ856058.1 | IGHV1S22*01 | tgtacaaga | tc | gaggagggg |  | ctacaatgattacgac | TRSRRGYNDYDGFAY |
| X59180.1 | IGHV1S22*01 | tgtacaaga |  | gaggagg |  | atgatggttac | TREEDDGYGAMDY |
| AY128954.1 | IGHV5-6-2*01 | tgtgcaagaca |  | gaggagg |  | gatggtta | ARQRRDGYYWYFDV |
| X59115.1 | IGHV8-8*01 | tgtgctcgaa |  | gagctggagg |  | gtatggtaactac | ARRAGGYGNYGWYFDV |
| DQ416713.1 | IGHV1-74*04 | tgtgcaa |  | gagctg |  | actatgatgacta | ARADYDDYDYFAFAY |
| AJ441120.1 | IGHV14-4*02 | tgtaatgc |  | gagaaggc |  | atgggtacta | NARRHGYYEMDY |
| AY229961.1 | IGHV1-20*02 | tgtgcaaga |  | gagaag |  | ggatacga | AREKGYEKYFDV |
| FJ751231.1 | IGHV5-12-2*01 | tgtgca |  | cgtcagacag |  | tctatgatggttactac | ARQTVYDGYYFTY |
| FJ751231.1 | IGHV5-12-2*01 | tgtgca |  | cgtcagacag |  | tctatgatggttactac | ARQTVYDGYYFTY |
| FJ751231.1 | IGHV5-12-2*01 | tgtgca |  | cgtcagacag |  | tctatgatggttactac | ARQTVYDGYYFTY |
| X99230.1 | IGHV1-80*01 | tgtgcaaga |  | cgggagac |  | tacgacggtag | ARRETTTVGRYYYAMDY |
| AF265688.1 | IGHV5-9-1*01 | tgtgcaag |  | caggggaggaa |  | atgatggt | ASRGGNDGYFDV |
| L21018.1 | IGHV5-6-3*01 | tgtgcaaga |  | cagagggggggaga |  | ctactatgattacgac | ARQRGGDYYDYDGYYFDY |
| L21018.1 | IGHV5-6-3*01 | tgtgcaaga |  | cagagggggggaga |  | ctactatgattacgac | ARQRGGDYYDYDGYYFDY |
| L21018.1 | IGHV5-6-3*01 | tgtgcaaga |  | cagagggggggaga |  | ctactatgattacgac | ARQRGGDYYDYDGYYFDY |
| L21016.1 | IGHV5-6-3*01 | tgtgcaaga |  | cagagggggggaga |  | ctactatgatcacgac | ARQRGGDYYDHDGYYFDY |
| L21016.1 | IGHV5-6-3*01 | tgtgcaaga |  | cagagggggggaga |  | ctactatgatcacgac | ARQRGGDYYDHDGYYFDY |
| L21016.1 | IGHV5-6-3*01 | tgtgcaaga |  | cagagggggggaga |  | ctactatgatcacgac | ARQRGGDYYDHDGYYFDY |
| D50382.1 | IGHV1-18*01 | tgtgcaaga | t | atggtttccaaaa |  | ttattatattttggac | ARYGFQNYYILDS |
| D50396.1 | IGHV1-18*01 | tgtgcaaga | t | atggtttccaaaa |  | ttactatattt | ARYGFQNYYILDS |
| AF240168.1 | IGHV9-4*02 | tgt |  | atgagatggg |  | attacgacggggg | MRWDYDGGFAY |
| AF240168.1 | IGHV9-4*02 | tgt |  | atgagatggg |  | attacgacggggg | MRWDYDGGFAY |
| D50378.1 | IGHV5-9-1*01 | tgt |  | ataagacgggatg |  | ataattccg | IRRDDNSGAWFAY |
| D50378.1 | IGHV5-9-1*01 | tgt |  | ataagacgggatg |  | ataattccg | IRRDDNSGAWFAY |
| AF178603.1 | IGHV1S135*01 | tgtgcaaga |  | aggggtaggaa |  | aactgggac | ARRGRKTGTGFAY |
| AF265687.1 | IGHV5-9-1*01 | tgtgcaaga |  | aggggaggaa |  | atgatggtt | ARRGGNDGCFDV |
| AF265686.1 | IGHV5-9-1*01 | tgttcaaga |  | aggggaggaa |  | atgatggt | SRRGGNDGYFDV |
| AF265685.1 | IGHV5-9-1*01 | tgtgcaaga |  | aggggaggaa |  | atgatggt | ARRGGNDGYFDV |
| AF265684.1 | IGHV5-9-1*01 | tgtacaaga |  | aggggaggaa |  | atgatggttgct | TRRGGNDGCLGV |
| AJ243908.1 | IGHV5-9-3*01 | tgtgcaagaca |  | agggacaggggtcgg |  | gtatggtaactac | ARQGTGVGYGNYNY |
| AM745099.1 | IGHV5-6*01 | tgtgcaaga |  | agaagtgaat |  | tttattactacggtaatacctac | ARRSEFYYYGNTYYYSAMDY |
| AM745099.1 | IGHV5-6*01 | tgtgcaaga |  | agaagtgaat |  | tttattactacggtaatacctac | ARRSEFYYYGNTYYYSAMDY |
| EU239745.1 | IGHV3-2*02 | tgtgcaaga |  | aggatat |  | tctatgatggttact | ARRIFYDGYYYFDY |
| M92385.1 | IGHV1-9*01 | tgt |  | acnagatgg |  | gggactgggacgg | TRWGTGTGHVMDY |
| AF546725.1 | IGHV2-2*02 | tgtgccagaaa |  | accgaaaacccctc |  | tctatgat | ARKPKTPLYDYAMDY |
| M92335.1 | IGHV5-12*02 | tgtacaagaca | t | accctttatgataccctt |  | tatggtaacta | TRHTLYDTLYGNYPVWFAD |
| AY878005.1 | IGHV5-17*03 | tgtgcaag |  | acagagagg | ga | tctactttgattacga | ARQRGIYFDYDKSWFAY |
| AY878005.1 | IGHV5-17*03 | tgtgcaag |  | acagagagg | ga | tctactttgattacga | ARQRGIYFDYDKSWFAY |
| AY878005.1 | IGHV5-17*03 | tgtgcaag |  | acagagagg | ga | tctactttgattacga | ARQRGIYFDYDKSWFAY |
| AY878005.1 | IGHV5-17*03 | tgtgcaag |  | acagagagg | ga | tctactttgattacga | ARQRGIYFDYDKSWFAY |
| DQ416706.1 | IGHV1-53*01 | tgt |  | ataagattgggc |  | tatttctacgg | IRLGYFYGDY |
| DQ416706.1 | IGHV1-53*01 | tgt |  | ataagattgggc |  | tatttctacgg | IRLGYFYGDY |
| AY172528.1 | IGHV1-9*01 | tgtgcaaga |  | aaggaaa |  | actataggtacg | ARKENYRYDWYFDV |
| AY172528.1 | IGHV1-9*01 | tgtgcaaga |  | aaggaaa |  | actataggtacg | ARKENYRYDWYFDV |
| AF044230.1 | IGHV1S135*01 | tgtgcaaga |  | aaagacct |  | ctactataggtacg | ARKDLYYRYXEWFAY |
| AF044230.1 | IGHV1S135*01 | tgtgcaaga |  | aaagacct |  | ctactataggtacg | ARKDLYYRYXEWFAY |
| AY369876.1 | IGHV1-26*01 | tgtg |  | tgagaggg |  | ggttact | VRGGYLGAMDY |
| AY369876.1 | IGHV1-26*01 | tgtg |  | tgagaggg |  | ggttact | VRGGYLGAMDY |
| GQ856056.1 | IGHV1-69*02 | tgtg |  | tgagatcg |  | gagggtaact | VRSEGNSMDY |
| GQ856056.1 | IGHV1-69*02 | tgtg |  | tgagatcg |  | gagggtaact | VRSEGNSMDY |
| AY174014.1 | IGHV2-9-1*01 | tgtgccagaga |  | ggaga |  | attactac | AREENYYFLYYAMDY |
| AY171991.1 | IGHV1-14*01 | tgtgcaaga |  | ggaaggg |  | actacggtagtag | ARGRDYGSRGYAMDY |
| AY171991.1 | IGHV1-14*01 | tgtgcaaga |  | ggaaggg |  | actacggtagtag | ARGRDYGSRGYAMDY |
| X01436.1 | IGHV2-2*02 | tgtgcc |  | gagatggggc |  | ctatggtggttac | AEMGPMVVTPFAY |
| AF023219.1 | IGHV2-6-7*01 | tgtgccagaga | t | gagaga |  | ggttacta | ARDERGYXXSSSFAY |
| AY171934.1 | IGHV3-2*02 | tgtgcaaga |  | gagaggggc | gaa | ttcattactacggct | ARERGEFITTAFDY |
| AY171934.1 | IGHV3-2*02 | tgtgcaaga |  | gagaggggc | gaa | ttcattactacggct | ARERGEFITTAFDY |
| AY171943.1 | IGHV1-9*01 | tgtgcaaga |  | gagagggg |  | aaatgg | ARERGNGYYAMDY |
| AY171943.1 | IGHV1-9*01 | tgtgcaaga |  | gagagggg |  | aaatgg | ARERGNGYYAMDY |
| AY835666.1 | IGHV10-1*02 | tgtgtgag |  | gagagg |  | gtcgatgcggt | VRRGSMRSAYYAMDY |
| AY835666.1 | IGHV10-1*02 | tgtgtgag |  | gagagg |  | gtcgatgcggt | VRRGSMRSAYYAMDY |
| DQ228207.1 | IGHV1-18*01 | tgtgcaag |  | gagagg |  | ggcagaggctac | ARRGAEATTWLAY |
| DQ228207.1 | IGHV1-18*01 | tgtgcaag |  | gagagg |  | ggcagaggctac | ARRGAEATTWLAY |
| AY172370.1 | IGHV1-9*01 | tgtgcaag |  | gagacggttttttgg |  | tattactacggtagtag | ARRRFFGITTVVDFDY |
| AY877974.1 | IGHV5-4*02 | tgtgcaag |  | gagac |  | tttat | ARRLYGLYAMDY |
| M19900.1 | IGHV9-4*02 | tgtgggag |  | gacag |  | attactacggtagta | GRTDYYGSTYYAMDY |
| AY172492.1 | IGHV1S16*01 | tgtacaa |  | gaaggaggag |  | ttactacggtagt | TRRRSYYGSGY |
| AY172492.1 | IGHV1S16*01 | tgtacaa |  | gaaggaggag |  | ttactacggtagt | TRRRSYYGSGY |
| AY172492.1 | IGHV1S16*01 | tgtacaa |  | gaaggaggag |  | ttactacggtagt | TRRRSYYGSGY |
| AY369898.1 | IGHV1-69*02 | tgtgcaaga |  | gaagga |  | gatggttac | AREGDGYYYAMDY |
| AY172871.1 | IGHV1-9*01 | tgtgcaaga |  | gaagga |  | attactacggtag | AREGITTVVDY |
| M24786.1 | IGHV1S45*01 | tgtgcaaga |  | gaagggg |  | gtttcggca | AREGGFGNSLDY |
| AY172003.1 | IGHV2-9-1*01 | tgtgccag |  | gaagggg |  | tatgattacgac | ARKGYDYDGGGWFAY |
| AY369954.1 | IGHV2-9-1*01 | tgtgccagag |  | gaaggcg |  | ctatgatggttactac | ARGRRYDGYYVDYFDY |
| X96755.1 | IGHV1-74*04 | tgtgcaa |  | gaagg |  | agctcgggct | ARRSSGYDFDY |
| L14741.1 | IGHV1-39*01 | tgtgcaag |  | gaagg |  | tacggctac | ARKVRLRYFDX |
| L08222.1 | IGHV8-8*01 | tgtg |  | gaagagc |  | tgggtacgac | GRAGYDRMDY |
| X59204.1 | IGHV1-14*01 | tgtgcaaga |  | gaagag |  | tatggttacg | AREEYGYGD |
| EF672198.1 | IGHV5-12-1*01 | tgtgcaaga |  | gaagaa |  | tactatggtaa | AREEYYGKAWFAY |
| EF672198.1 | IGHV5-12-1*01 | tgtgcaaga |  | gaagaa |  | tactatggtaa | AREEYYGKAWFAY |
| AY128972.1 | IGHV7-1*02 | tgttca |  | cgaga |  | ttatttctacggtggt | SRDYFYGGGWYFDV |
| U55401.1 | IGHV1-9*01 | tgtgc |  | ccagtccct |  | ctatggtaact | AQSLYGNYY |
| AY182575.1 | IGHV2-9*01 | tgtgc |  | cagagatc |  | attactacggtagtag | ARDHYYGSSWFAY |
| AY182575.1 | IGHV2-9*01 | tgtgc |  | cagagatc |  | attactacggtagtag | ARDHYYGSSWFAY |
| X94416.1 | IGHV3-2*02 | tgtgcaag |  | cagaagcc |  | tctactatggccacga | ASRSLYYGHEGFGY |
| X94416.1 | IGHV3-2*02 | tgtgcaag |  | cagaagcc |  | tctactatggccacga | ASRSLYYGHEGFGY |
| AY182581.1 | IGHV2-9*01 | tgtgc |  | cagaa |  | attactacgg | ARNYYGYFDY |
| HQ015085.1 | IGHV5-6*01 | tgtgcaa |  | caaggggag |  | atggtaact | ATRGDGNYFFDY |
| AY173965.1 | IGHV2-9-1*01 | tgtgcca |  | caaatcctcc |  | ttattactacggtagtagc | ATNPPYYYGSSYDAMDY |
| AY182573.1 | IGHV2-9*01 | tgtgc |  | caaac |  | attactacggtagtagctac | AKHYYGSSYDY |
| AJ878860.1 | IGHV1-12*01 | tgtgcaaga |  | agtgac | a | tctactatggtaactac | ARSDIYYGNYNALDY |
| AB121075.1 | IGHV1-18*01 | cgtgcaaga |  | aggagggg |  | ctactatgattacgac | ARRRGYYDYDGAWFAY |
| AY853690.1 | IGHV5-6*01 | tgtgcaagaca |  | aggagggcg |  | ttactacggtagtag | ARQGGRYYGSRHAMDY |
| AY877958.1 | IGHV1-26*01 | tgtgcaaga | tct | aggaggg |  | gttacgacagtt | ARSRRGYDSYYYAMDY |
| EU934847.1 | IGHV1-67*01 | tgtgcaaga |  | aggaaga |  | aactgggac | ARRKKLGQGYAMDY |
| AY174004.1 | IGHV3-2*02 | tgtgcaaga |  | aggaagggc | a | tctatgatggtt | ARRKGIYDGYYFDY |
| AY174004.1 | IGHV3-2*02 | tgtgcaaga |  | aggaagggc | a | tctatgatggtt | ARRKGIYDGYYFDY |
| AY182488.1 | IGHV2-2*02 | tgtgccagaaa |  | aggaat |  | ctactataggtatta | ARKGIYYRYYYAXXY |
| AF546739.1 | IGHV1-4*01 | tgtgcaaga |  | agatgg |  | gattacgac | ARRWDYDGDY |
| AY877978.1 | IGHV5-9*04 | tgtgcaagac |  | agagggga |  | attattacgg | ARQRGIITDLGHFDY |
| AY878006.1 | IGHV5-17*03 | tgtacaag |  | agaggga |  | tactacggtagtagcta | TREGYYGSSYWFAY |
| AF118949.1 | IGHV2-9-1*01 | tgtgccagaga | t | agagg |  | gtatggtaac | ARDRGYGNYYAMDY |
| AY877957.1 | IGHV1-69*02 | tgtgcaaga |  | agagg |  | ctacggtagtagct | ARRGYGSSYYYAMDY |
| AF023214.1 | IGHV5-12-2*01 | tgt |  | acgagac |  | atcgtggt | TRHRGSMDY |
| AF023214.1 | IGHV5-12-2*01 | tgt |  | acgagac |  | atcgtggt | TRHRGSMDY |
| AY878001.1 | IGHV5-17*03 | tgtgcaag |  | acgagg |  | ttactacggtag | ARRGYYGSAMDY |
| AY878000.1 | IGHV5-17*03 | tgtgcaag |  | acgag |  | attactacggtag | ARRDYYGSAMDY |
| U01241.1 | IGHV5-6-2*01 | tgtg |  | acagac |  | gggacagctcg | DRRDSSFSLYFDY |
| U01241.1 | IGHV5-6-2*01 | tgtg |  | acagac |  | gggacagctcg | DRRDSSFSLYFDY |
| AY171935.1 | IGHV14-3*02 | tgtgcta |  | aagga |  | tactacggtagta | AKGYYGSIFDY |
| M97874.1 | IGHV1S135*01 | tgtacaa |  | aagagggggg |  | gattgcg | TKEGGIAGFAY |
| AY594679.1 | IGHV2-9*02 | tgtgtaaga |  | aagagg |  | ggtaactac | VRKRGNYLYYAMDY |
| X86536.1 | IGHV2-5*01 | tgtgccaaaaa |  | aagagg |  | gattacg | AKKRGITVDYYVMDY |
| D55698.1 | IGHV1-9*01 | tgtgcaa |  | aaaccg |  | attactacgctagtggctac | AKTDYYASGYGFDY |

| **C57BL/6** |  |  |  |  |  |  |  |
| --- | --- | --- | --- | --- | --- | --- | --- |
| **Accession #** | **V_H_ Gene** | **V_H_** | **P** | **N1^a^** | **P** | **D_H_** | **CDR3 (aa)^b^** |
| AB589314.1 | IGHV1-72*01 | tgtgc |  | tggataccg |  | attgggacg | AGYRLGRFFDN |
| Z12806.1 | IGHV1-72*01 | tgtg |  | tcggagaggaggac |  | tactacggtagtaact | VGEEDYYGSNSRFAY |
| Z12806.1 | IGHV1-72*01 | tgtg |  | tcggagaggaggac |  | tactacggtagtaact | VGEEDYYGSNSRFAY |
| Z12806.1 | IGHV1-72*01 | tgtg |  | tcggagaggaggac |  | tactacggtagtaact | VGEEDYYGSNSRFAY |
| Z12806.1 | IGHV1-72*01 | tgtg |  | tcggagaggaggac |  | tactacggtagtaact | VGEEDYYGSNSRFAY |
| AB160676.1 | IGHV1-78*01 | tgt |  | tcgagag |  | agaacgattacta | SRENDYYNMDY |
| AF455949.1 | IGHV1-62-3*01 | tgtgcaa |  | tcagga |  | attactacgggagtagc | AIRNYYGSSYDAMDY |
| AB160144.1 | IGHV1-62-3*01 | tgtgcaaga |  | gtggaagaa |  | tacggtagtagctac | ARVEEYGSSYVGDH |
| AB160144.1 | IGHV1-62-3*01 | tgtgcaaga |  | gtggaagaa |  | tacggtagtagctac | ARVEEYGSSYVGDH |
| AB187281.1 | IGHV1-72*01 | tgtgcaag |  | gtacgag |  | tactacggccac | ARYEYYGHYYFDY |
| AB589305.1 | IGHV1-72*01 | tgttca |  | ggaggaaga |  | tatggtatct | SGGRYGIFHFDY |
| AB160674.1 | IGHV1-72*01 | tgtgcgaga |  | ggaggaa |  | attactacgctagtagc | ARGGNYYASSLDY |
| AB589128.1 | IGHV1-72*01 | tgtgc |  | gcgataca |  | attactacggtggtag | ARYNYYGGSYFDY |
| JF690661.1 | IGHV1-82*01 | tgtgcaaga |  | gccaagggga |  | attactacggtagtagc | ARAKGNYYGSSPLDY |
| AB160811.1 | IGHV1-9*01 | tgtgcaaga |  | gaggggaga |  | acagctcaggc | AREGRTAQALYYFDY |
| AB516488.1 | IGHV1-72*01 | tgtgcaaga |  | gaaagag |  | gatggttactac | ARERGWLLRFAY |
| AB516488.1 | IGHV1-72*01 | tgtgcaaga |  | gaaagag |  | gatggttactac | ARERGWLLRFAY |
| AB043394.1 | IGHV1-72*01 | tgtgcaag |  | gggaag |  | actggg | ARGRLGLHFDY |
| AF065352.1 | IGHV1-72*01 | tgtgcgaga |  | ggagagg |  | attactacggtagt | ARGEDYYGSAFGY |
| AF065352.1 | IGHV1-72*01 | tgtgcgaga |  | ggagagg |  | attactacggtagt | ARGEDYYGSAFGY |
| AF065352.1 | IGHV1-72*01 | tgtgcgaga |  | ggagagg |  | attactacggtagt | ARGEDYYGSAFGY |
| AB516550.1 | IGHV1-72*01 | tgtg |  | ggagag |  | gggggta | GRGGYFDF |
| AB516550.1 | IGHV1-72*01 | tgtg |  | ggagag |  | gggggta | GRGGYFDF |
| AB043329.1 | IGHV1-72*01 | tgtgcaaga |  | ggacagt |  | attcttaccgc | ARGQYSYRHFDY |
| M27623.1 | IGHV14-2*01 | tgtgctaga |  | gctaga |  | tattactacggtagtag | ARARYYYGSSYFDY |
| M27619.1 | IGHV1-72*01 | tgtgcaaga |  | gcaagg |  | tattactacggtagtag | ARARYYYGSSYFDY |
| M27620.1 | IGHV1-72*01 | tgtgcaaga |  | gcaaga |  | tattactacggtagtagc | ARARYYYGSSYFDV |
| AB052128.1 | IGHV1-55*01 | tgtgcaaga |  | gaggag |  | attactacggt | AREEITTVHYFDY |
| X80958.1 | IGHV1-81*01 | tgtgcaaga |  | gagaaag |  | actacgatagtagc | AREKDYDSSAWFAC |
| X80976.1 | IGHV1-81*01 | tgtgcaaga |  | gagaaag |  | actacgatactagc | AREKDYDTSAWFAF |
| JF690665.1 | IGHV1-81*01 | tgtgcaaga |  | gaagaactacgggag |  | tactactttgactac | AREELREYYFDYW |
| JF690665.1 | IGHV1-81*01 | tgtgcaaga |  | gaagaactacgggag |  | tactactttgactac | AREELREYYFDYW |
| AJ240314.1 | IGHV1-74*01 | tgtgcaata |  | gaaaga |  | aactgggac | AIERNWDVYFDY |
| AF458178.1 | IGHV1-59*01 | tgtgcaag |  | cttccccccaaac |  | tattactacggtagtagc | ASFPPNYYYGSSGTY |
| AB069868.1 | IGHV1-72*01 | tgt |  | cgaagata |  | ctactatggtaact | RRYYYGNFINY |
| AB069868.1 | IGHV1-72*01 | tgt |  | cgaagata |  | ctactatggtaact | RRYYYGNFINY |
| AB589216.1 | IGHV1-72*01 | tgtgcaaga |  | cccccgaaaagccctcctc |  | acggctcaggctac | ARPPKSPPHGSGYQFAY |
| AB589131.1 | IGHV1-72*01 | tgtgc |  | ccgatatag |  | ttactacggtgttggt | ARYSYYGVGFFDY |
| AB589120.1 | IGHV1-72*01 | tgtgc |  | ccgatatag |  | ttactacggtggtag | ARYSYYGGSFFDY |
| JF690646.1 | IGHV1-61*01 | tgtgc |  | ccgaaggaggggt |  | tactatggtaactac | ARRRGYYGNYVGYAMDY |
| JF690646.1 | IGHV1-61*01 | tgtgc |  | ccgaaggaggggt |  | tactatggtaactac | ARRRGYYGNYVGYAMDY |
| JF690646.1 | IGHV1-61*01 | tgtgc |  | ccgaaggaggggt |  | tactatggtaactac | ARRRGYYGNYVGYAMDY |
| AJ427328.1 | IGHV1-74*01 | tgtgcaata | t | cggagattcgcgg |  | gtatggta | AISEIRGYGNYVDY |
| AJ427328.1 | IGHV1-74*01 | tgtgcaata | t | cggagattcgcgg |  | gtatggta | AISEIRGYGNYVDY |
| AB517321.1 | IGHV1-72*01 | tgtaca |  | cgatacg |  | aactgggac | TRYELGRYFDV |
| AB067782.1 | IGHV1-72*01 | tgtgc |  | caagagg |  | agtaactac | AKRSNYGAFDV |
| AB067782.1 | IGHV1-72*01 | tgtgc |  | caagagg |  | agtaactac | AKRSNYGAFDV |
| AY114409.1 | IGHV1-72*01 | tgtgcaaga | t | attgtgcaaga |  | tattactacggtagtag | ARYCARYYYGSSFDY |
| AB159929.1 | IGHV1-55*01 | tgtgcaaga |  | atggggagaat |  | tgatggt | ARMGRIDGNFDY |
| AB159929.1 | IGHV1-55*01 | tgtgcaaga |  | atggggagaat |  | tgatggt | ARMGRIDGNFDY |
| AB516597.1 | IGHV1-72*01 | tgtgcaaga | t | atggcaaat |  | cggtagta | ARYGKSVVPWYFDV |
| AJ240335.1 | IGHV1-50*01 | tgtgcaaga |  | agcgggaagg |  | aactgggac | ARSGKELGRARYYFDY |
| AJ240335.1 | IGHV1-50*01 | tgtgcaaga |  | agcgggaagg |  | aactgggac | ARSGKELGRARYYFDY |
| AB159861.1 | IGHV1-64*01 | tgtgcaa |  | agaaagacggccc |  | ttactatgg | AKKDGPYYGMEY |
| AB159861.1 | IGHV1-64*01 | tgtgcaa |  | agaaagacggccc |  | ttactatgg | AKKDGPYYGMEY |
| AB159861.1 | IGHV1-64*01 | tgtgcaa |  | agaaagacggccc |  | ttactatgg | AKKDGPYYGMEY |
| AB516525.1 | IGHV1-72*01 | tgtgcaaga | t | actatccgaga |  | tattactat | ARYYPRYYYTLDY |
| AB057864.1 | IGHV1-72*01 | tgtgcaaga | t | acgatat |  | tagtaactac | ARYDISNYIGFAY |
| AB517255.1 | IGHV1-72*01 | tgtgcaaga | t | atgagc |  | attactccggtag | ARYEHYSGRGAFDY |
| AB574259.1 | IGHV1-72*01 | tgtgcgaga | t | acgagag |  | actggga | ARYERLGGSNYFDY |
| AB574259.1 | IGHV1-72*01 | tgtgcgaga | t | acgagag |  | actggga | ARYERLGGSNYFDY |
| AB571405.1 | IGHV1-72*01 | tgtgcaa |  | aataca |  | attactacggtggtag | AKYNYYGGSYFDY |
| AB057950.1 | IGHV1-72*01 | tgtgcaaga |  | aaggagg |  | gatggttcctac | ARKEGWFLHFAY |
| AB057950.1 | IGHV1-72*01 | tgtgcaaga |  | aaggagg |  | gatggttcctac | ARKEGWFLHFAY |
| AB160783.1 | IGHV1-55*01 | tgtgcaaga |  | aaggag |  | ctgggac | ARKELGRYWYFDV |
| AB160783.1 | IGHV1-55*01 | tgtgcaaga |  | aaggag |  | ctgggac | ARKELGRYWYFDV |
| AB159889.1 | IGHV1-9*01 | tgtgcaaga | t | tgagaac |  | ctttagtacttat | ARLRTFSTYYFDY |
| AB159889.1 | IGHV1-9*01 | tgtgcaaga | t | tgagaac |  | ctttagtacttat | ARLRTFSTYYFDY |
| AB160806.1 | IGHV1-53*01 | tgtgcaaga |  | ggaga |  | ggggact | ARGEGTFDY |
| AB030762.1 | IGHV1-72*01 | tgtgcaaga |  | ggaaga |  | tatggtg | ARGRYGVHFDY |
| AB030760.1 | IGHV1-72*01 | tgttcaaga |  | ggaaggtt |  | tggttacg | SRGRFGYAHFDY |
| AB030760.1 | IGHV1-72*01 | tgttcaaga |  | ggaaggtt |  | tggttacg | SRGRFGYAHFDY |
| AB043443.1 | IGHV1-72*01 | tgtgcaaga |  | ggaagg |  | actggg | ARGRTGPFDY |
| AB043443.1 | IGHV1-72*01 | tgtgcaaga |  | ggaagg |  | actggg | ARGRTGPFDY |
| AB043290.1 | IGHV1-72*01 | tgtgcaaga |  | ggaagg |  | attactacggtagtag | ARGRITTVVVHFDY |
| AB043290.1 | IGHV1-72*01 | tgtgcaaga |  | ggaagg |  | attactacggtagtag | ARGRITTVVVHFDY |
| AF065379.1 | IGHV1-72*01 | tgtgcaaga |  | ggaag |  | gtttggta | ARGRFGNHFDY |
| AY648635.1 | IGHV5-9-3*01 | tgtgcaaga |  | gatgctaatcacc |  | acttgagatccg | ARDANHHLRSDAMDY |
| AJ240331.1 | IGHV1-74*01 | tgtgcaat |  | gagagggta |  | tagtagc | AMRGYSSGYYFDY |
| AJ240331.1 | IGHV1-74*01 | tgtgcaat |  | gagagggta |  | tagtagc | AMRGYSSGYYFDY |
| AB517226.1 | IGHV1-72*01 | tgtgcaaga |  | gagaggggt |  | tggttact | ARERGWLLHAMDY |
| AB517226.1 | IGHV1-72*01 | tgtgcaaga |  | gagaggggt |  | tggttact | ARERGWLLHAMDY |
| AB070336.1 | IGHV1-74*01 | tgtgcaata |  | gagaggggt |  | tatgg | AIERGYGYYAMDY |
| AB070336.1 | IGHV1-74*01 | tgtgcaata |  | gagaggggt |  | tatgg | AIERGYGYYAMDY |
| AF455991.1 | IGHV1-80*01 | tgtgcaaga |  | gagagggc |  | cttactagg | ARERALLGYFDY |
| AF455991.1 | IGHV1-80*01 | tgtgcaaga |  | gagagggc |  | cttactagg | ARERALLGYFDY |
| Z12765.1 | IGHV1-52*01 | tgtgcaaga |  | gagag |  | ctactatggtaactac | ARESYYGNYGAMDY |
| Z12803.1 | IGHV1-72*01 | tgtacaag |  | gagag |  | actatgattacgac | TRRDYDYDPFDV |
| AB160569.1 | IGHV1-9*01 | tgtgcaaga |  | gacagggg |  | ctacggtagtagc | ARDRGYGSSAFAY |
| AF455929.1 | IGHV1-63*01 | tgtgcaag |  | gaaggatccgggaccttc |  | cggtagtagct | ARKDPGPSGSSSFDY |
| AJ240333.1 | IGHV1-55*01 | tgtgcaaga |  | gaagggggc |  | gattacgac | AREGGDYDDY |
| AB052124.1 | IGHV1-64*01 | tgtgcaag |  | gaagggcc |  | attactacggtagtag | ARKGHYYGSSFDY |
| AB159893.1 | IGHV1-50*01 | tgtgcaaga |  | gaaggg |  | agtggt | AREGSGYFDY |
| AB069864.1 | IGHV1-53*01 | tgtacaag |  | gaagg |  | attactacggtagtag | TRKDYYGSSGFVY |
| AB517477.1 | IGHV1-72*01 | tgtgcaaga | tc | cgaag |  | ggttactac | ARSEGLLRFAY |
| JF690653.1 | IGHV2-9*02 | tgtg |  | ccagagg |  | cgggct | ARGGLPYRHFDY |
| JF690653.1 | IGHV2-9*02 | tgtg |  | ccagagg |  | cgggct | ARGGLPYRHFDY |
| JF690616.1 | IGHV2-9*02 | tgtg |  | ccagaaaactctctct |  | ctatggtaa | ARKLSLYGNLDY |
| AB517244.1 | IGHV1-72*01 | tgtgcaaga |  | caggaccga |  | acggta | ARQDRTVQFAY |
| AB043330.1 | IGHV1-72*01 | tgtacaa |  | caggac |  | agtatgcttac | TTGQYAYRHFDY |
| AF458160.1 | IGHV5-4*01 | tgtgcaaga |  | cagagtcc |  | ctatgattacgac | ARQSPYDYDVSFDY |
| AJ833583.1 | IGHV14-4*01 | tgtac |  | cagacgcc |  | tttattactacggtagtagc | TRRLYYYGSSLDY |
| AB057940.1 | IGHV1-72*01 | tgtgcaag |  | caaggggc |  | atataagta | ASKGHISNYFDY |
| AB163931.1 | IGHV1-72*01 | tgtgc |  | caaggg |  | aactgggac | AKGTGTFAY |
| X07721.1 | IGHV1-72*03 | g |  | caaga |  | tatatttacg | RYIYGYYFDY |
| JF690647.1 | IGHV2-9*01 | tgtgc |  | caaacacgg |  | tggttact | AKHGGYSTLYAMDY |
| AB516623.1 | IGHV1-72*01 | tgtgcaaga | tc | aggagagg |  | ggttactac | ARSGEGLLRFAY |
| AB516623.1 | IGHV1-72*01 | tgtgcaaga | tc | aggagagg |  | ggttactac | ARSGEGLLRFAY |
| AB516623.1 | IGHV1-72*01 | tgtgcaaga | tc | aggagagg |  | ggttactac | ARSGEGLLRFAY |
| AY114428.1 | IGHV1-62-3*01 | tgtgcaaga |  | aggaggggatgg |  | gattacg | ARRRGWDYGFAY |
| AB589266.1 | IGHV1-72*01 | tgtgtaaga | tc | aggaat |  | ctatgattacgac | VRSGIYDYDDAWFVY |
| AB517360.1 | IGHV1-72*01 | tgtgcaaga |  | agaggg |  | tatgattacgac | ARRGYDYDGMFAY |
| AF455987.1 | IGHV1-18*01 | tgtgcaaga |  | agaggacat |  | gatacgccggg | ARRGHDTPGAMDY |
| AB164009.1 | IGHV1-72*01 | tgtgcaaga |  | agaga |  | tccgcag | ARRDPQTYAMDY |
| AJ240437.1 | IGHV1-55*01 | tgtgcaaga |  | agaagggc |  | ctatgatggt | ARRRAYDGLDY |
| AY114426.1 | IGHV1-72*01 | tgtgcaaga | t | acgagtt |  | cagctcaggctac | ARYEFSSGYGFDY |
| AB043299.1 | IGHV1-72*01 | tgtgcaaga | t | acgaggg |  | tactacggtagtagc | ARYEGTTVVAFDY |
| AB517231.1 | IGHV1-72*01 | tgtgcaaga | t | acgagg |  | gattacg | ARYEGLRAWFAY |
| AB571459.1 | IGHV1-72*01 | tgtgcaaga | t | acgag |  | tacttcggtagaag | ARYEYFGRSYFDY |
| AB069880.1 | IGHV1-72*01 | tgt |  | acgag |  | ttattactacggtagtagc | TSYYYGSSVFDY |
| AB160427.1 | IGHV1-47*01 | tgtgcaag |  | acgag |  | gacagctcaggctac | ARRGQLRLRRYFDY |
| AB159966.1 | IGHV1-69*02 | tgtgcaaga |  | aagaccc |  | atgatggtttctac | ARKTHDGFYSYAMDF |
| JF690660.1 | IGHV6-3*01 | tgcaca |  | aagacgg |  | tttttactacgg | TKTVFTTGDY |

| **NZB/NZW** |  |  |  |  |  |  |  |
| --- | --- | --- | --- | --- | --- | --- | --- |
| **Accession #** | **V_H_ Gene** | **V_H_** | **P** | **N1^a^** | **P** | **D_H_** | **CDR3 (aa)^b^** |
| AF093561.1 | IGHV5-6-2*01 | tgtacaagac |  | gtgatgcc |  | ctgagctgc | TRRDALSCLYFDV |
| AF093563.1 | IGHV5-6-2*01 | tg |  | gacaagacggg |  | atggtaact | TRRDGNSYLYFDV |
| Z22071.1 | IGHV1-19*01 | tgtgcaaga |  | ggagag |  | actacggtagtag | ARGETTVVGKGYYFDY |
| Z22071.1 | IGHV1-19*01 | tgtgcaaga |  | ggagag |  | actacggtagtag | ARGETTVVGKGYYFDY |
| AF321941.1 | IGHV2-6*03 | tgtgccaga |  | ccctcga |  | tacgg | ARPSIRGYFDY |
| AF321951.1 | IGHV2-6*01 | tgt |  | actagaccg |  | ggtttct | TRPGFFYAMDY |
| AF321951.1 | IGHV2-6*01 | tgt |  | actagaccg |  | ggtttct | TRPGFFYAMDY |
| U55552.1 | IGHV3-3*01 | tgtgcgagag |  | ggagacgaa |  | agtatggt | ARGRRKYGLYYAMDY |
| U55525.1 | IGHV1-39*01 | tgtgcaaga |  | gagaggggga |  | agtatggtaa | ARERGKYGNHV |
| U55525.1 | IGHV1-39*01 | tgtgcaaga |  | gagaggggga |  | agtatggtaa | ARERGKYGNHV |
| U55568.1 | IGHV5-12-2*01 | tgtacaaga |  | gagaggga |  | ttattacttcggcagt | TRERDYYFGSGRLLDY |
| U55568.1 | IGHV5-12-2*01 | tgtacaaga |  | gagaggga |  | ttattacttcggcagt | TRERDYYFGSGRLLDY |
| M37013.1 | IGHV1-82*01 | tgtgcaaga |  | gagagg |  | actgggac | ARERTGTGFVY |
| M37013.1 | IGHV1-82*01 | tgtgcaaga |  | gagagg |  | actgggac | ARERTGTGFVY |
| U55469.1 | IGHV2-9*02 | tgtg |  | ccagtc |  | attactacggtactagct | ASHYYGTSSWYXDV |
| U55518.1 | IGHV2-9*02 | tgtg |  | ccagtc |  | attactacggtactagct | ASHYYGTSSWYFDV |
| U55510.1 | IGHV2-9*02 | tgtg |  | ccagaaacacnccc |  | ctgggac | ARNTPLGRRYYFDY |
| U55510.1 | IGHV2-9*02 | tgtg |  | ccagaaacacnccc |  | ctgggac | ARNTPLGRRYYFDY |
| U55468.1 | IGHV2-9*02 | tgtg |  | ccagaagggaact |  | ctacggtagtagcta | ARRELYGSSYGWYFDV |
| U55468.1 | IGHV2-9*02 | tgtg |  | ccagaagggaact |  | ctacggtagtagcta | ARRELYGSSYGWYFDV |
| U55484.1 | IGHV2-9*02 | tgtg |  | ccagaaccctct |  | acagctcgggctac | ARTLYSSGYSWFAY |
| U55484.1 | IGHV2-9*02 | tgtg |  | ccagaaccctct |  | acagctcgggctac | ARTLYSSGYSWFAY |
| Z30962.1 | IGHV2-9*02 | tgtg |  | ccaga |  | gatggttact | ARDGYSFFDY |
| U60455.1 | IGHV1-14*01 | tgtgcaaga |  | aggag |  | agggacg | ARRRGTYYFDY |
| U55572.1 | IGHV5-17*01 | tgtgtaagg |  | aagagga |  | attacga | VRKRNYDTMDY |
| Z22138.1 | IGHV1-84*01 | tgtgcaa |  | aagagg |  | actgggac | AKEDWDGXFVY |

| **NZM2410** |  |  |  |  |  |  |  |
| --- | --- | --- | --- | --- | --- | --- | --- |
| **Accession #** | **V_H_ Gene** | **V_H_** | **P** | **N1^a^** | **P** | **D_H_** | **CDR3 (aa)^b^** |
| AY436929.1 | IGHV1-5*01 | tgta |  | aaggag |  | atgatggttactac | KGDDGYYPFYFDY |
| AY436929.1 | IGHV1-5*01 | tgta |  | aaggag |  | atgatggttactac | KGDDGYYPFYFDY |
| AY436937.1 | IGHV2-9*02 | tgtg |  | ccagaggtc |  | tctactatggttacga | ARGLYYGYEAWFAY |
| AY436937.1 | IGHV2-9*02 | tgtg |  | ccagaggtc |  | tctactatggttacga | ARGLYYGYEAWFAY |
| AY436946.1 | IGHV2-9*02 | tgtg |  | ccaga |  | aaagagg | ARKEEFGWFAY |
| AY437066.1 | IGHV1-42*01 | tgtgcaaga |  | agagg |  | ttacttgggggg | ARRGYLGGALDY |
| AY436917.1 | IGHV1-63*01 | tgtgcaaga |  | aaaagg |  | ggttacg | ARKRGYAPNAY |

| **NZW/BXSB** |  |  |  |  |  |  |  |
| --- | --- | --- | --- | --- | --- | --- | --- |
| **Accession #** | **V_H_ Gene** | **V_H_** | **P** | **N1^a^** | **P** | **D_H_** | **CDR3 (aa)^b^** |
| AF019944.1 | IGHV5-9-3*01 | tgtgcaagac |  | gggggggcaaggga |  | tactttgactac | ARRGGKGYFDYFDV |
| U41432.1 | IGHV1-9*01 | tgtgcacga |  | agatggagtaaaag |  | actttactggt | ARRWSKRLYWSFHV |
| U41432.1 | IGHV1-9*01 | tgtgcacga |  | agatggagtaaaag |  | actttactggt | ARRWSKRLYWSFHV |

| **NZW/SWR** |  |  |  |  |  |  |  |
| --- | --- | --- | --- | --- | --- | --- | --- |
| **Accession #** | **V_H_ Gene** | **V_H_** | **P** | **N1^a^** | **P** | **D_H_** | **CDR3 (aa)^b^** |
| X75245.1 | IGHV5-9-3*01 | tgtg |  | tcagaccgaggga |  | ctactctaataac | VRPRDYSNNVYYFDN |
| X75245.1 | IGHV5-9-3*01 | tgtg |  | tcagaccgaggga |  | ctactctaataac | VRPRDYSNNVYYFDN |
| X75245.1 | IGHV5-9-3*01 | tgtg |  | tcagaccgaggga |  | ctactctaataac | VRPRDYSNNVYYFDN |
| X75221.1 | IGHV5-16*01 | tgtgcaaga |  | ccagta |  | gggacaactcgggc | ARPVGTTRARGAWFAY |
| X75224.1 | IGHV5-16*01 | tgtgcacga |  | ccagta |  | gggacaactcgggc | ARPVGTTRARGAWFAY |

| **C57BL/6/lpr** |  |  |  |  |  |  |  |
| --- | --- | --- | --- | --- | --- | --- | --- |
| **Accession #** | **V_H_ Gene** | **V_H_** | **P** | **N1^a^** | **P** | **D_H_** | **CDR3 (aa)^b^** |
| AB043208.1 | IGHV1-72*01 | tgtgcaaga |  | gggaag |  | tacggtattagc | ARGKYGISHFDH |
| AB043173.1 | IGHV1-72*01 | tgt |  | accagag |  | gacagctcgggct | TRGQLGLHFDC |
| AB043224.1 | IGHV1-72*01 | tgtg |  | tgagagg |  | ctactttggta | VRGYFGNHFDY |
| AB043224.1 | IGHV1-72*01 | tgtg |  | tgagagg |  | ctactttggta | VRGYFGNHFDY |
| AB043170.1 | IGHV1-72*01 | tgtg |  | tgaga |  | tattactacggtcgttgc | VRYYYGRCLDY |
| AB043160.1 | IGHV1-72*02 | g |  | caaga |  | tattactacgctggtag | RYYYAGSFDS |
| AB043221.1 | IGHV1-72*01 | tgt |  | acgaga |  | tattactacggtggt | TRYYYGGLFDS |

| **MRL/lpr** |  |  |  |  |  |  |  |
| --- | --- | --- | --- | --- | --- | --- | --- |
| **Accession #** | **V_H_ Gene** | **V_H_** | **P** | **N1^a^** | **P** | **D_H_** | **CDR3 (aa)^b^** |
| GQ240580.1 | IGHV1-39*01 | tgtacaaga |  | gggaaga |  | agacagctcgggct | TRGKKTARAAFFVY |
| U28342.1 | IGHV1-14*01 | tgtgcaaga | tc | gagaactcct | g | cctattatagtaacta | ARSRTPAYYSNYPWFAY |
| X64998.1 | IGHV1-69*02 | tgtgcaaga |  | cgggaatattacg | a | tttacgacgaggg | ARREYYDLRRGHAMDY |
| X65001.1 | IGHV1-69*02 | tgttcaaga |  | cggagat |  | attacgac | SRRRYYDLRRGYAMDY |
| X65001.1 | IGHV1-69*02 | tgttcaaga |  | cggagat |  | attacgac | SRRRYYDLRRGYAMDY |
| AF289176.1 | IGHV5-9*04 | tgtgcaagac | g | aggagcg |  | tatagtaa | ARRGAYSKGFAY |
| AF289176.1 | IGHV5-9*04 | tgtgcaagac | g | aggagcg |  | tatagtaa | ARRGAYSKGFAY |
| L08975.1 | IGHV1-82*01 | tgtgcaaga |  | agaagaagaa |  | attactatggtg | ARRRRNYYGDYYSMDY |
| L08975.1 | IGHV1-82*01 | tgtgcaaga |  | agaagaagaa |  | attactatggtg | ARRRRNYYGDYYSMDY |
| L08975.1 | IGHV1-82*01 | tgtgcaaga |  | agaagaagaa |  | attactatggtg | ARRRRNYYGDYYSMDY |
| L08975.1 | IGHV1-82*01 | tgtgcaaga |  | agaagaagaa |  | attactatggtg | ARRRRNYYGDYYSMDY |
| L08975.1 | IGHV1-82*01 | tgtgcaaga |  | agaagaagaa |  | attactatggtg | ARRRRNYYGDYYSMDY |
| U21061.1 | IGHV2-6*01 | tgt |  | actagag |  | atgatggttactac | TRDDGYYGMDY |
| X65535.1 | IGHV2-2*01 | tgtgccagaa |  | taagatct |  | tatggtaactac | ARIRSYGNYKPWFAY |
| X65535.1 | IGHV2-2*01 | tgtgccagaa |  | taagatct |  | tatggtaactac | ARIRSYGNYKPWFAY |
| X65004.1 | IGHV1-14*01 | tgtgcaaga |  | aagagggt |  | ctataataactac | ARKRVYNNYVLRSSLYAMDY |
| X60333.1 | IGHV1-14*01 | tgtgcaaga |  | aagagtag |  | tagactacgcagt | ARKSSRLRSTLDY |
| X60334.1 | IGHV1-14*01 | tgtgcaaga |  | aagagtag |  | taggctacgcagt | ARKSSRLRSTLDY |

| **SLE1** |  |  |  |  |  |  |  |
| --- | --- | --- | --- | --- | --- | --- | --- |
| **Accession #** | **V_H_ Gene** | **V_H_** | **P** | **N1^a^** | **P** | **D_H_** | **CDR3 (aa)^b^** |
| AF455287.1 | IGHV1-55*01 | tgtgcaaga |  | gaagctagacc |  | ctctagtaact | AREARPSSNSLPHYAMDY |
| AF455287.1 | IGHV1-55*01 | tgtgcaaga |  | gaagctagacc |  | ctctagtaact | AREARPSSNSLPHYAMDY |
| AF455287.1 | IGHV1-55*01 | tgtgcaaga |  | gaagctagacc |  | ctctagtaact | AREARPSSNSLPHYAMDY |
| AF459904.1 | IGHV1-9*01 | tgtgcaag |  | gaggaga |  | aactggg | ARRRNWDYFDY |
| AF455302.1 | IGHV1-50*01 | tgtgcaaga |  | gaggaggg |  | gacagctcaggc | AREEGTAQAAWFAY |
| AF455317.1 | IGHV1-72*01 | tgtgcaaga |  | caggagcaagacc |  | tgattacg | ARQEQDLITGAY |
| AF455317.1 | IGHV1-72*01 | tgtgcaaga |  | caggagcaagacc |  | tgattacg | ARQEQDLITGAY |
| AY454478.1 | IGHV1-64*01 | tg |  | cacaagaagcaccct |  | gtatggta | TRSTLYGILCS |
| AY454478.1 | IGHV1-64*01 | tg |  | cacaagaagcaccct |  | gtatggta | TRSTLYGILCS |
| AY454515.1 | IGHV1-82*01 | tgtgcaaga | tc | cgaaggg |  | ggttac | ARSEGGYRFAY |
| AY454515.1 | IGHV1-82*01 | tgtgcaaga | tc | cgaaggg |  | ggttac | ARSEGGYRFAY |
| AF459858.1 | IGHV1-26*01 | tgtgcaaga |  | acccgaatg |  | tatgattacgac | ARTRMYDYDAYYYAMDY |
| AF459879.1 | IGHV1-39*01 | tgtgcaaga | tc | gatgccc |  | attactacggtagtag | ARSMPITTVVEYYFDY |
| AY454488.1 | IGHV1-81*01 | tgtgcaaga |  | gagaggg |  | gattacgac | ARERGLRRYFDV |
| AY454488.1 | IGHV1-81*01 | tgtgcaaga |  | gagaggg |  | gattacgac | ARERGLRRYFDV |
| AY454511.1 | IGHV2-9*02 | tgtg |  | ccagaac |  | ctatagtaactac | ARTYSNYRTMDY |
| AY454511.1 | IGHV2-9*02 | tgtg |  | ccagaac |  | ctatagtaactac | ARTYSNYRTMDY |
| AY454509.1 | IGHV3-6*01 | tgtgcaagag |  | caggaa |  | attactacggtagt | ARAGNYYGSAYFDY |
| AF455354.1 | IGHV1-81*01 | tgtgcaaga |  | agaggg |  | attactacggtagtagctac | ARRGITTVVATGDY |
| AY454524.1 | IGHV1-64*01 | tgtgcaaga |  | agaaacct |  | gtatggta | ARRNLYGTLDY |
| AY454524.1 | IGHV1-64*01 | tgtgcaaga |  | agaaacct |  | gtatggta | ARRNLYGTLDY |
| AY454491.1 | IGHV5-17*01 | tgtgcaag |  | aaaaatg |  | gctatcctc | ARKMAILHYYSMDY |

| **SLE1/SLE3** |  |  |  |  |  |  |  |
| --- | --- | --- | --- | --- | --- | --- | --- |
| **Accession #** | **V_H_ Gene** | **V_H_** | **P** | **N1^a^** | **P** | **D_H_** | **CDR3 (aa)^b^** |
| AY437081.1 | IGHV1-12*01 | tgt |  | actagaaagagaggatt |  | acggcgaggg | TRKRGLRRGCFDV |
| AY437081.1 | IGHV1-12*01 | tgt |  | actagaaagagaggatt |  | acggcgaggg | TRKRGLRRGCFDV |
| AY437081.1 | IGHV1-12*01 | tgt |  | actagaaagagaggatt |  | acggcgaggg | TRKRGLRRGCFDV |
| AY437081.1 | IGHV1-12*01 | tgt |  | actagaaagagaggatt |  | acggcgaggg | TRKRGLRRGCFDV |
| AY437081.1 | IGHV1-12*01 | tgt |  | actagaaagagaggatt |  | acggcgaggg | TRKRGLRRGCFDV |
| AY437059.1 | IGHV1-22*01 | tgtgcaaga |  | ccaaaccc |  | ttattactacggtagtagct | ARPNPYYYGSSFLDY |
| AY437078.1 | IGHV1-42*01 | tgtgcaaga |  | agaaa |  | aaccgggac | ARRKTGTFFDF |
| AY437063.1 | IGHV1-12*01 | tgtacaaga |  | aagagag |  | gattgcg | TRKRGLRRGYFDV |
| AY437069.1 | IGHV1-12*01 | tgtgcaaga |  | aagagag |  | gattacg | ARKRGLRRGYFDV |
| AY437080.1 | IGHV1-12*01 | tgtgcaaga |  | aagagag |  | gattgcg | ARKRGLRRGFFDV |

| **NOD/NOR** |  |  |  |  |  |  |  |
| --- | --- | --- | --- | --- | --- | --- | --- |
| **Accession #** | **V_H_ Gene** | **V_H_** | **P** | **N1^a^** | **P** | **D_H_** | **CDR3 (aa)^b^** |
| EU568228.1 | IGHV13-2*01 | tgtagtag |  | gggggaaaga | a | tctactatgattacgac | SRGERIYYDYDGDAMDY |
| EU568234.1 | IGHV1-39*01 | tgtgcaaga |  | agaaga | a | tctactatgattacga | ARRRIYYDYEVYAMDY |
| EU568234.1 | IGHV1-39*01 | tgtgcaaga |  | agaaga | a | tctactatgattacga | ARRRIYYDYEVYAMDY |
| EU568226.1 | IGHV5-9*01 | tgtgcaaga |  | caaggg |  | tactacggt | ARQGYYGPKDY |

| **Auto Ab** |  |  |  |  |  |  |  |
| --- | --- | --- | --- | --- | --- | --- | --- |
| **Accession #** | **V_H_ Gene** | **V_H_** | **P** | **N1^a^** | **P** | **D_H_** | **CDR3 (aa)^b^** |
| EU568228.1 | IGHV13-2*01 | tgtagtag |  | gggggaaaga | a | tctactatgattacgac | SRGERIYYDYDGDAMDY |
| AF289176.1 | IGHV5-9*04 | tgtgcaagac | g | aggagcg |  | tatagtaa | ARRGAYSKGFAY |
| AF289176.1 | IGHV5-9*04 | tgtgcaagac | g | aggagcg |  | tatagtaa | ARRGAYSKGFAY |
| EU568234.1 | IGHV1-39*01 | tgtgcaaga |  | agaaga | a | tctactatgattacga | ARRRIYYDYEVYAMDY |
| EU568234.1 | IGHV1-39*01 | tgtgcaaga |  | agaaga | a | tctactatgattacga | ARRRIYYDYEVYAMDY |
| M20829.1 | IGHV10S4*01 | tgtgtgaga |  | gatgct | g | ctaactgg | VRDAANWSAWFAY |
| EU568226.1 | IGHV5-9*01 | tgtgcaaga |  | caaggg |  | tactacggt | ARQGYYGPKDY |

| **Anti-DNA Ab** |  |  |  |  |  |  |  |
| --- | --- | --- | --- | --- | --- | --- | --- |
| **Accession #** | **V_H_ Gene** | **V_H_** | **P** | **N1^a^** | **P** | **D_H_** | **CDR3 (aa)^b^** |
| Z22071.1 | IGHV1-19*01 | tgtgcaaga |  | ggagag |  | actacggtagtag | ARGETTVVGKGYYFDY |
| Z22071.1 | IGHV1-19*01 | tgtgcaaga |  | ggagag |  | actacggtagtag | ARGETTVVGKGYYFDY |
| L14733.1 | IGHV1-74*01 | tgtgcaa |  | gaggagaatatggtaa |  | ctacgtttattactata | ARGEYGNYVYYYTMHY |
| L14733.1 | IGHV1-74*01 | tgtgcaa |  | gaggagaatatggtaa |  | ctacgtttattactata | ARGEYGNYVYYYTMHY |
| L14733.1 | IGHV1-74*01 | tgtgcaa |  | gaggagaatatggtaa |  | ctacgtttattactata | ARGEYGNYVYYYTMHY |
| U28342.1 | IGHV1-14*01 | tgtgcaaga | tc | gagaactcct | g | cctattatagtaacta | ARSRTPAYYSNYPWFAY |
| AY454478.1 | IGHV1-64*01 | tg |  | cacaagaagcaccct |  | gtatggta | TRSTLYGILCS |
| AY454478.1 | IGHV1-64*01 | tg |  | cacaagaagcaccct |  | gtatggta | TRSTLYGILCS |
| AY454515.1 | IGHV1-82*01 | tgtgcaaga | tc | cgaaggg |  | ggttac | ARSEGGYRFAY |
| AY454515.1 | IGHV1-82*01 | tgtgcaaga | tc | cgaaggg |  | ggttac | ARSEGGYRFAY |
| AF178603.1 | IGHV1S135*01 | tgtgcaaga |  | aggggtaggaa |  | aactgggac | ARRGRKTGTGFAY |
| AF289176.1 | IGHV5-9*04 | tgtgcaagac | g | aggagcg |  | tatagtaa | ARRGAYSKGFAY |
| AF289176.1 | IGHV5-9*04 | tgtgcaagac | g | aggagcg |  | tatagtaa | ARRGAYSKGFAY |
| AY437081.1 | IGHV1-12*01 | tgt |  | actagaaagagaggatt |  | acggcgaggg | TRKRGLRRGCFDV |
| AY437081.1 | IGHV1-12*01 | tgt |  | actagaaagagaggatt |  | acggcgaggg | TRKRGLRRGCFDV |
| AY437081.1 | IGHV1-12*01 | tgt |  | actagaaagagaggatt |  | acggcgaggg | TRKRGLRRGCFDV |
| AY437081.1 | IGHV1-12*01 | tgt |  | actagaaagagaggatt |  | acggcgaggg | TRKRGLRRGCFDV |
| AY437081.1 | IGHV1-12*01 | tgt |  | actagaaagagaggatt |  | acggcgaggg | TRKRGLRRGCFDV |
| AY436929.1 | IGHV1-5*01 | tgta |  | aaggag |  | atgatggttactac | KGDDGYYPFYFDY |
| AY436929.1 | IGHV1-5*01 | tgta |  | aaggag |  | atgatggttactac | KGDDGYYPFYFDY |
| U55552.1 | IGHV3-3*01 | tgtgcgagag |  | ggagacgaa |  | agtatggt | ARGRRKYGLYYAMDY |
| U55525.1 | IGHV1-39*01 | tgtgcaaga |  | gagaggggga |  | agtatggtaa | ARERGKYGNHV |
| U55525.1 | IGHV1-39*01 | tgtgcaaga |  | gagaggggga |  | agtatggtaa | ARERGKYGNHV |
| U55568.1 | IGHV5-12-2*01 | tgtacaaga |  | gagaggga |  | ttattacttcggcagt | TRERDYYFGSGRLLDY |
| U55568.1 | IGHV5-12-2*01 | tgtacaaga |  | gagaggga |  | ttattacttcggcagt | TRERDYYFGSGRLLDY |
| AY454488.1 | IGHV1-81*01 | tgtgcaaga |  | gagaggg |  | gattacgac | ARERGLRRYFDV |
| AY454488.1 | IGHV1-81*01 | tgtgcaaga |  | gagaggg |  | gattacgac | ARERGLRRYFDV |
| L14741.1 | IGHV1-39*01 | tgtgcaag |  | gaagg |  | tacggctac | ARKVRLRYFDX |
| L08222.1 | IGHV8-8*01 | tgtg |  | gaagagc |  | tgggtacgac | GRAGYDRMDY |
| U55469.1 | IGHV2-9*02 | tgtg |  | ccagtc |  | attactacggtactagct | ASHYYGTSSWYXDV |
| U55518.1 | IGHV2-9*02 | tgtg |  | ccagtc |  | attactacggtactagct | ASHYYGTSSWYFDV |
| AY436937.1 | IGHV2-9*02 | tgtg |  | ccagaggtc |  | tctactatggttacga | ARGLYYGYEAWFAY |
| AY436937.1 | IGHV2-9*02 | tgtg |  | ccagaggtc |  | tctactatggttacga | ARGLYYGYEAWFAY |
| U55510.1 | IGHV2-9*02 | tgtg |  | ccagaaacacnccc |  | ctgggac | ARNTPLGRRYYFDY |
| U55510.1 | IGHV2-9*02 | tgtg |  | ccagaaacacnccc |  | ctgggac | ARNTPLGRRYYFDY |
| U55468.1 | IGHV2-9*02 | tgtg |  | ccagaagggaact |  | ctacggtagtagcta | ARRELYGSSYGWYFDV |
| U55468.1 | IGHV2-9*02 | tgtg |  | ccagaagggaact |  | ctacggtagtagcta | ARRELYGSSYGWYFDV |
| U55484.1 | IGHV2-9*02 | tgtg |  | ccagaaccctct |  | acagctcgggctac | ARTLYSSGYSWFAY |
| U55484.1 | IGHV2-9*02 | tgtg |  | ccagaaccctct |  | acagctcgggctac | ARTLYSSGYSWFAY |
| AY454511.1 | IGHV2-9*02 | tgtg |  | ccagaac |  | ctatagtaactac | ARTYSNYRTMDY |
| AY454511.1 | IGHV2-9*02 | tgtg |  | ccagaac |  | ctatagtaactac | ARTYSNYRTMDY |
| AY436946.1 | IGHV2-9*02 | tgtg |  | ccaga |  | aaagagg | ARKEEFGWFAY |
| Z30962.1 | IGHV2-9*02 | tgtg |  | ccaga |  | gatggttact | ARDGYSFFDY |
| AY437059.1 | IGHV1-22*01 | tgtgcaaga |  | ccaaaccc |  | ttattactacggtagtagct | ARPNPYYYGSSFLDY |
| AY454509.1 | IGHV3-6*01 | tgtgcaagag |  | caggaa |  | attactacggtagt | ARAGNYYGSAYFDY |
| AY437066.1 | IGHV1-42*01 | tgtgcaaga |  | agagg |  | ttacttgggggg | ARRGYLGGALDY |
| AY454524.1 | IGHV1-64*01 | tgtgcaaga |  | agaaacct |  | gtatggta | ARRNLYGTLDY |
| AY454524.1 | IGHV1-64*01 | tgtgcaaga |  | agaaacct |  | gtatggta | ARRNLYGTLDY |
| AY437078.1 | IGHV1-42*01 | tgtgcaaga |  | agaaa |  | aaccgggac | ARRKTGTFFDF |
| U55572.1 | IGHV5-17*01 | tgtgtaagg |  | aagagga |  | attacga | VRKRNYDTMDY |
| Z22138.1 | IGHV1-84*01 | tgtgcaa |  | aagagg |  | actgggac | AKEDWDGXFVY |
| AY437063.1 | IGHV1-12*01 | tgtacaaga |  | aagagag |  | gattgcg | TRKRGLRRGYFDV |
| AY437069.1 | IGHV1-12*01 | tgtgcaaga |  | aagagag |  | gattacg | ARKRGLRRGYFDV |
| AY437080.1 | IGHV1-12*01 | tgtgcaaga |  | aagagag |  | gattgcg | ARKRGLRRGFFDV |
| AY436917.1 | IGHV1-63*01 | tgtgcaaga |  | aaaagg |  | ggttacg | ARKRGYAPNAY |
| AY454491.1 | IGHV5-17*01 | tgtgcaag |  | aaaaatg |  | gctatcctc | ARKMAILHYYSMDY |

| **ANA Ab** |  |  |  |  |  |  |  |
| --- | --- | --- | --- | --- | --- | --- | --- |
| **Accession #** | **V_H_ Gene** | **V_H_** | **P** | **N1^a^** | **P** | **D_H_** | **CDR3 (aa)^b^** |
| AY454478.1 | IGHV1-64*01 | tg |  | cacaagaagcaccct |  | gtatggta | TRSTLYGILCS |
| AY454478.1 | IGHV1-64*01 | tg |  | cacaagaagcaccct |  | gtatggta | TRSTLYGILCS |
| AY454515.1 | IGHV1-82*01 | tgtgcaaga | tc | cgaaggg |  | ggttac | ARSEGGYRFAY |
| AY454515.1 | IGHV1-82*01 | tgtgcaaga | tc | cgaaggg |  | ggttac | ARSEGGYRFAY |
| AY437081.1 | IGHV1-12*01 | tgt |  | actagaaagagaggatt |  | acggcgaggg | TRKRGLRRGCFDV |
| AY437081.1 | IGHV1-12*01 | tgt |  | actagaaagagaggatt |  | acggcgaggg | TRKRGLRRGCFDV |
| AY437081.1 | IGHV1-12*01 | tgt |  | actagaaagagaggatt |  | acggcgaggg | TRKRGLRRGCFDV |
| AY437081.1 | IGHV1-12*01 | tgt |  | actagaaagagaggatt |  | acggcgaggg | TRKRGLRRGCFDV |
| AY437081.1 | IGHV1-12*01 | tgt |  | actagaaagagaggatt |  | acggcgaggg | TRKRGLRRGCFDV |
| AY436929.1 | IGHV1-5*01 | tgta |  | aaggag |  | atgatggttactac | KGDDGYYPFYFDY |
| AY436929.1 | IGHV1-5*01 | tgta |  | aaggag |  | atgatggttactac | KGDDGYYPFYFDY |
| AY454488.1 | IGHV1-81*01 | tgtgcaaga |  | gagaggg |  | gattacgac | ARERGLRRYFDV |
| AY454488.1 | IGHV1-81*01 | tgtgcaaga |  | gagaggg |  | gattacgac | ARERGLRRYFDV |
| AY436937.1 | IGHV2-9*02 | tgtg |  | ccagaggtc |  | tctactatggttacga | ARGLYYGYEAWFAY |
| AY436937.1 | IGHV2-9*02 | tgtg |  | ccagaggtc |  | tctactatggttacga | ARGLYYGYEAWFAY |
| AY454511.1 | IGHV2-9*02 | tgtg |  | ccagaac |  | ctatagtaactac | ARTYSNYRTMDY |
| AY454511.1 | IGHV2-9*02 | tgtg |  | ccagaac |  | ctatagtaactac | ARTYSNYRTMDY |
| AY436946.1 | IGHV2-9*02 | tgtg |  | ccaga |  | aaagagg | ARKEEFGWFAY |
| AY437059.1 | IGHV1-22*01 | tgtgcaaga |  | ccaaaccc |  | ttattactacggtagtagct | ARPNPYYYGSSFLDY |
| AY454509.1 | IGHV3-6*01 | tgtgcaagag |  | caggaa |  | attactacggtagt | ARAGNYYGSAYFDY |
| AY437066.1 | IGHV1-42*01 | tgtgcaaga |  | agagg |  | ttacttgggggg | ARRGYLGGALDY |
| AY454524.1 | IGHV1-64*01 | tgtgcaaga |  | agaaacct |  | gtatggta | ARRNLYGTLDY |
| AY454524.1 | IGHV1-64*01 | tgtgcaaga |  | agaaacct |  | gtatggta | ARRNLYGTLDY |
| AY437078.1 | IGHV1-42*01 | tgtgcaaga |  | agaaa |  | aaccgggac | ARRKTGTFFDF |
| AY437063.1 | IGHV1-12*01 | tgtacaaga |  | aagagag |  | gattgcg | TRKRGLRRGYFDV |
| AY437069.1 | IGHV1-12*01 | tgtgcaaga |  | aagagag |  | gattacg | ARKRGLRRGYFDV |
| AY437080.1 | IGHV1-12*01 | tgtgcaaga |  | aagagag |  | gattgcg | ARKRGLRRGFFDV |
| AY436917.1 | IGHV1-63*01 | tgtgcaaga |  | aaaagg |  | ggttacg | ARKRGYAPNAY |
| AY454491.1 | IGHV5-17*01 | tgtgcaag |  | aaaaatg |  | gctatcctc | ARKMAILHYYSMDY |

| **Anti-NP Ab** |  |  |  |  |  |  |  |
| --- | --- | --- | --- | --- | --- | --- | --- |
| **Accession #** | **V_H_ Gene** | **V_H_** | **P** | **N1^a^** | **P** | **D_H_** | **CDR3 (aa)^b^** |
| AJ240314.1 | IGHV1-74*01 | tgtgcaata |  | gaaaga |  | aactgggac | AIERNWDVYFDY |
| AY078478.1 | IGHV3-8*01 | tg |  | cacaagatggg |  | atgttaagtac | TRWDVKYGLAY |
| AY078478.1 | IGHV3-8*01 | tg |  | cacaagatggg |  | atgttaagtac | TRWDVKYGLAY |
| AY078478.1 | IGHV3-8*01 | tg |  | cacaagatggg |  | atgttaagtac | TRWDVKYGLAY |
| AB067782.1 | IGHV1-72*01 | tgtgc |  | caagagg |  | agtaactac | AKRSNYGAFDV |
| AB067782.1 | IGHV1-72*01 | tgtgc |  | caagagg |  | agtaactac | AKRSNYGAFDV |
| AJ240335.1 | IGHV1-50*01 | tgtgcaaga |  | agcgggaagg |  | aactgggac | ARSGKELGRARYYFDY |
| AJ240335.1 | IGHV1-50*01 | tgtgcaaga |  | agcgggaagg |  | aactgggac | ARSGKELGRARYYFDY |
| M18570.1 | IGHV6-3*01 | tgcaca |  | acgaagg |  | gatggttactac | TTKGWLLPFVY |
| M18570.1 | IGHV6-3*01 | tgcaca |  | acgaagg |  | gatggttactac | TTKGWLLPFVY |
| AB030762.1 | IGHV1-72*01 | tgtgcaaga |  | ggaaga |  | tatggtg | ARGRYGVHFDY |
| AB030760.1 | IGHV1-72*01 | tgttcaaga |  | ggaaggtt |  | tggttacg | SRGRFGYAHFDY |
| AB030760.1 | IGHV1-72*01 | tgttcaaga |  | ggaaggtt |  | tggttacg | SRGRFGYAHFDY |
| AJ240331.1 | IGHV1-74*01 | tgtgcaat |  | gagagggta |  | tagtagc | AMRGYSSGYYFDY |
| AJ240331.1 | IGHV1-74*01 | tgtgcaat |  | gagagggta |  | tagtagc | AMRGYSSGYYFDY |
| AJ240333.1 | IGHV1-55*01 | tgtgcaaga |  | gaagggggc |  | gattacgac | AREGGDYDDY |
| AJ240437.1 | IGHV1-55*01 | tgtgcaaga |  | agaagggc |  | ctatgatggt | ARRRAYDGLDY |

^a^ The identified V_H_ replacement footprints are highlighted in *red* in the N1 regions.

^b^ The amino acids encoded by the identified V_H_ replacement footprints are highlighted in *red* in the amino acid sequences of the CDR3 regions.

^c.^One sequence with multiple positions of potential VH replacement footprints are listed in a box.
